# Association of CYP2D6 genotype and tamoxifen metabolites with breast cancer recurrence in a low-dose trial

**DOI:** 10.1038/s41523-021-00236-6

**Published:** 2021-03-25

**Authors:** Andrea DeCensi, Harriet Johansson, Thomas Helland, Matteo Puntoni, Debora Macis, Valentina Aristarco, Silvia Caviglia, Tania Buttiron Webber, Irene Maria Briata, Mauro D’Amico, Davide Serrano, Aliana Guerrieri-Gonzaga, Ersilia Bifulco, Steinar Hustad, Håvard Søiland, Luca Boni, Bernardo Bonanni, Gunnar Mellgren

**Affiliations:** 1 Division of Medical Oncology, E.O. Galliera Hospital, Genoa, Italy; 2 Division of Cancer Prevention and Genetics, IEO, European Institute of Oncology IRCCS, Milan, Italy; 3 Hormone Laboratory, Department of Medical Biochemistry and Pharmacology, Haukeland University Hospital, Bergen, Norway; 4 Clinical Trial Unit, Office of the Scientific Director, E.O. Galliera Hospital, Genoa, Italy; 5 Department of Clinical Science, University of Bergen, Bergen, Norway; 6 Department of Breast and Endocrine Surgery, Stavanger University Hospital, Stavanger, Norway; 7 IRCCS San Martino Hospital, Genoa, Italy

**Keywords:** Breast cancer, Cancer prevention

## Abstract

Low-dose tamoxifen halves recurrence in non-invasive breast cancer without significant adverse events. Some adjuvant trials with tamoxifen 20 mg/day had shown an association between low endoxifen levels (9–16 nM) and recurrence, but no association with CYP2D6 was shown in the NSABP P1 and P2 prevention trials. We studied the association of CYP2D6 genotype and tamoxifen metabolites with tumor biomarkers and recurrence in a randomized phase III trial of low-dose tamoxifen. Median (IQR) endoxifen levels at year 1 were 8.4 (5.3–11.4) in patients who recurred vs 7.5 (5.1–10.2) in those who did not recur (*p* = 0.60). Tamoxifen and metabolites significantly decreased C-reactive protein (CRP, *p* < 0.05), and a CRP increase after 3 years was associated with higher risk of recurrence (HR = 4.37, 95% CI, 1.14–16.73, *P* = 0.03). In conclusion, endoxifen is below 9 nM in most subjects treated with 5 mg/day despite strong efficacy and there is no association with recurrence, suggesting that the reason for tamoxifen failure is not poor drug metabolism. Trial registration: ClinicalTrials.gov, Identifier: NCT01357772.

Tamoxifen is the standard of care in women at high risk for breast cancer, including women with pre-invasive disorders, but toxicity, especially menopausal symptoms and rare serious adverse events such as endometrial cancer and venous thromboembolism have largely hampered its uptake in clinical practice^1^. We conducted a phase-III de-escalation trial in women with operated hormone-sensitive or unknown breast intraepithelial neoplasia who were randomized to either low dose tamoxifen, 5 mg/day, or placebo for 3 years. After a median follow-up of 5.1 years (IQR, 3.9–6.3), low dose tamoxifen significantly decreased recurrence by 52% and contralateral breast events by 75%^2^. Serious adverse events, including endometrial cancer and venous thromboembolic events, and patient-reported outcomes were not different between arms except for less than one extra hot flash per day on tamoxifen, thus providing a new treatment option in the management of breast intraepithelial neoplasia. A secondary analysis showed that the effect was non significantly greater in peri/postmenopausal women^3^. Some retrospective studies with tamoxifen 20 mg/day have shown an association between poor CYP2D6 metabolizers or levels of the most potent metabolite endoxifen below 9 or 16 nM and higher risk of recurrence in the adjuvant setting^4–6^, but the association with CYP2D6 has not been shown in the NSABP P1 and P2 tamoxifen prevention trials^7^. We assessed the *CYP2D6* genotype and measured tamoxifen metabolites to determine their association with (1) menopausal symptoms, (2) liver mediated biomarkers of drug estrogenicity associated with breast cancer recurrence, including IGF-I, SHBG, and C-reactive protein (CRP)^8^, and (3) risk of recurrence.

The participant flow diagram is depicted in Supplementary Fig. 1. Endoxifen concentrations were linearly associated with CYP2D6 metabolizer status (*p* < 0.001, Supplementary Fig. 2). Median (IQR) endoxifen levels were 8.4 (5.2–11.3) and 8.8 (5.8–11.5) at 1 and 3 years, with only 42% and 47% of subjects reaching 9 nM. Using the threshold of 16 nM identified by Madlensky et al.^6^, only 6% and 10% reached this level. Median endoxifen levels were related to pill count (4.3, 7.4 and 9.0 nM for medication possession rate <80%, 80–99.9%, 100%, respectively, *p*-trend = 0.001). Median (IQR) endoxifen levels at year 1 were 7.5 (5.1–10.2) in the 7 patients who recurred vs 8.4 (5.3–11.4) in the 156 patients who did not recur (Wilcoxon rank-sum test *p* = 0.6); 4 events were observed in women with endoxifen <8.4 nM (median value) compared to 3 events in women ≥8.4 nM (log-rank *p* = 0.74, Fig. 1). A similar pattern was noted for 4OH-tamoxifen (Supplementary Fig. 3). There was no association between metabolite levels and menopausal symptoms, such as hot flashes or vaginal dryness/discharge, nor between *CYP2D6* status and biomarker changes (not shown). The associations between tamoxifen and endoxifen concentrations and IGF-I, SHBG, and CRP levels at 1 year are summarized in Table 1. Tamoxifen, endoxifen and 4OH-tamoxifen levels were associated with a significant decrease of IGF-I levels at 1 year in peri-/postmenopausal women, whereas no significant change was observed in premenopausal women (*p*-interaction with menopause = 0.04, 0.20, and 0.14, respectively). Only tamoxifen levels were positively associated with SHBG increase in peri-/postmenopausal women (*p*-interaction with menopause = 0.04). Tamoxifen, endoxifen and 4OH-tamoxifen were associated with a significant decrease of CRP. Noticeably, an increase in CRP after 3 years predicted a high risk of recurrence (HR = 4.37, 95% CI, 1.14–16.73, *P* = 0.03) compared to women with no increase of CRP (Fig. 2).

**Fig. 1:**
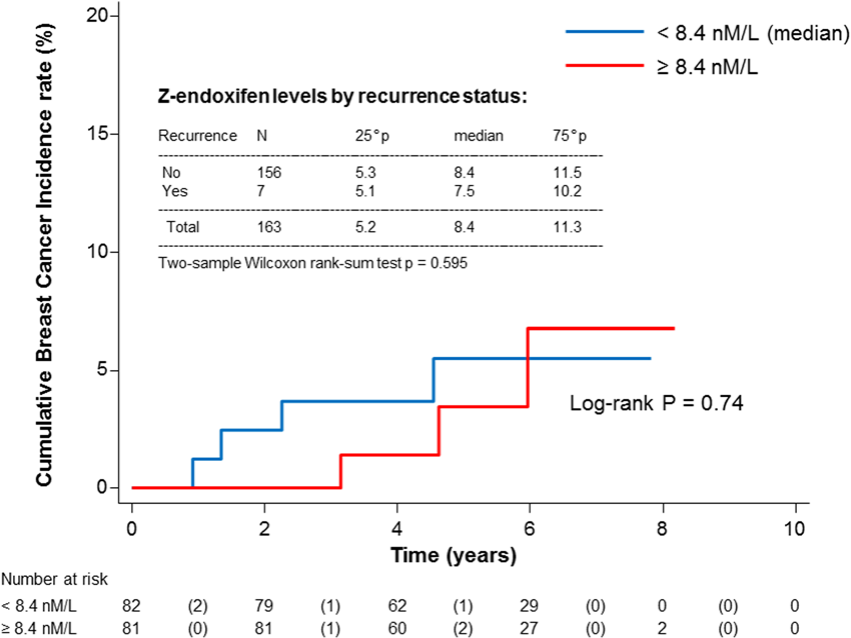
Cumulative breast cancer recurrence curves in the tamoxifen arm according to Z-endoxifen, nmol/L and by recurrence status (yes/no). Red line represents subjects with endoxifen level above the median value, the blue line below the median value (log-rank *p* = 0.74).

**Table 1:** Associations between tamoxifen and endoxifen and 4OH-tamoxifen concentrations and biomarker levels at 1 year.

	Tamoxifen	Endoxifen	Z-4OHtam
IGF-1			
All women	*n* = 154	*n* = 153	*n* = 153
	−0.110^a^ (0.038)	−0.096^a^ (0.067)	−0.088^a^ (0.091)
Pre-menopause	*n* = 56	*n* = 56	*n* = 56
	−0.086 (0.422)	−0.003 (0.979)	0.042 (0.687)
Peri/post-menopause	*n* = 98	*n* = 97	*n* = 97
	−0.179 (0.005)	−0.198 (0.002)	−8.873 (0.001)
SHBG			
All women	*n* = 154	*n* = 153	*N* = 153
	0.100^a^ (0.101)	0.045 (0.460)	0.100 (0.183)
Pre-menopause	*n* = 56		—
	−0.048 (0.627)	—	
Peri/post-menopause	*n* = 98		—
	0.172 (0.009)	—	
CRP^b^			
All women	*n* = 154	*n* = 153	*N* = 153
	−0.182 (0.007)	−0.136 (0.038)	−0.255 (0.014)
Pre-menopause	—	—	—
Peri/post-menopause	—	—	—

Data are beta standardized coefficients (*p*-values in parentheses) of biomarker parameters from linear regression models with biomarker level as response variable, metabolite level as explanatory, adjusting for age, medication possession rate at 1 year, BMI, menopausal status, and biomarker level at baseline.

^a^*p* interactions between biomarker level and menopausal status at baseline: ≤0.2; p-interaction for tamoxifen, endoxifen and 4OH-tamoxifen with menopause on IGF-I = 0.04, 0.20 and 0.14, respectively; p-interaction for tamoxifen with menopause on SHBG = 0.04

^b^CRP was log-transformed in linear regression models.

Note: Coefficients were calculated also in subgroups by menopausal status when *p* for interaction ≤0.2.

**Fig. 2:**
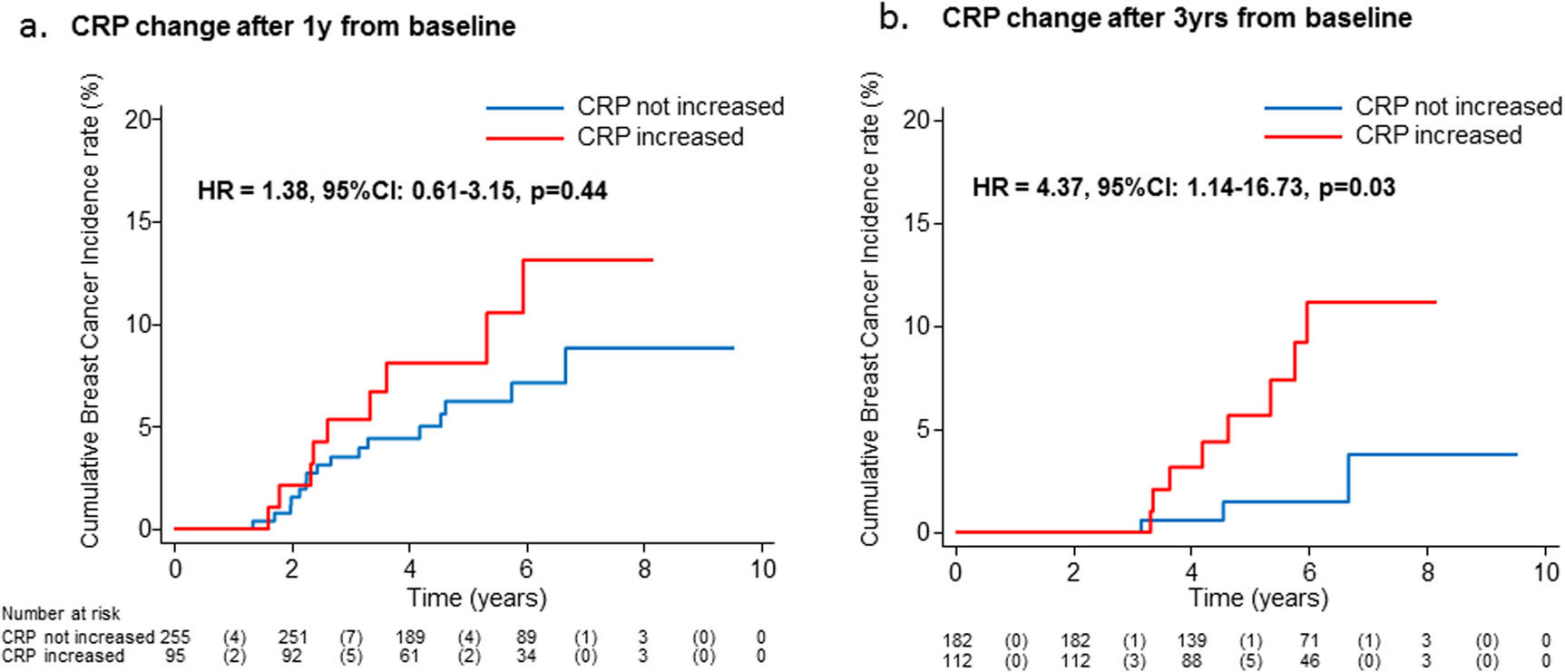
Cumulative breast cancer recurrence according to C-reactive protein increase. Results are shown irrespective of treatment allocation at 1 year (**a**) and 3 years (**b**). Hazard ratios were adjusted for age, BMI, and treatment arm.

Our findings indicate that most of the patients treated with 5 mg/day of tamoxifen do not attain circulating levels of endoxifen above 9 nmol/L, and 10% or less attain endoxifen levels of 16 nmol/L, which have been considered minimal thresholds of efficacy in retrospective analyses of adjuvant studies using 20 mg/day of tamoxifen^5,6^. Internal validity was confirmed by the strong correlation between CYP2D6 genotype or medication compliance and endoxifen levels. In contrast to previous retrospective studies^5,6^, in our prospective study there was no difference in endoxifen or 4OH-tamoxifen levels between patients who recurred versus those who did not, although our conclusions are based on only 7 events and so they are admittedly underpowered. Differences with a prior retrospective study using the same laboratory are the patient population, main outcome measure, and length of follow-up, which was recurrence after non-invasive disease at 5.1 years in our study versus breast cancer mortality from invasive disease after 14 years in the retrospective study^5^. Despite these limitations, our results suggest that even very low levels of endoxifen are effective and seem to discount the hypothesis that the reason for tamoxifen failure is poor drug metabolism. In line with this view, recent attempts to define minimal threshold levels of efficacy for endoxifen and the CYP2D6 genotype with full dose tamoxifen have been unsuccessful in prospective studies using recurrence as the main outcome measure^9^. Moreover, in poor/intermediate metabolizers due to CYP2D6-variant alleles, increasing tamoxifen dosing did not achieve a higher PFS rate in a randomized trial^10^. Finally, while there have been some studies demonstrating an association between CYP2D6 metabolism and/or endoxifen levels in patients with invasive cancer^4–6,11^, the one study evaluating CYP2D6 metabolism and outcomes in the NSABP P1/P2 clinical trials demonstrated no association between CYP2D6 metabolism and the risk of an invasive/non-invasive breast cancer in subjects taking tamoxifen 20 mg/day for primary prevention^7^. The mechanisms of the apparently greater effect of low Z-endoxifen in the preventive setting versus the adjuvant setting might be related to the higher ER beta expression in normal breast relative to invasive breast cancer^12^ and the high sensitivity of ER-beta to endoxifen^13^, so that low endoxifen concentrations are still sufficient to exert a preventive effect in ER-beta rich tissue.

Our study indicates that 5 mg/day of tamoxifen favorably modulates three liver mediated biomarkers of drug estrogenicity^8^ which have been associated with increased risk of recurrence and may at least in part explain the large antitumor effect of low dose tamoxifen observed in peri/postmenopausal women^3^. First, in peri-/postmenopausal women IGF-I was 0.18 and 0.20 standard deviations lower for every standard deviation increase of tamoxifen and endoxifen concentrations, respectively. While the main antitumor effect of tamoxifen is attributed to the much higher binding affinity of endoxifen to ER, the liver mediated effect of tamoxifen may also contribute per se to the drug efficacy given the association between IGF-I levels and breast cancer risk^14^ or recurrence^15^. Second, SHBG increased with increasing tamoxifen levels in peri/postmenopausal women. Notably, high levels of SHBG have been shown to be inversely associated with breast cancer risk in large meta-analyses of prospective studies^16^. Moreover, we have previously shown in a different trial that an increase of SHBG during low dose tamoxifen was associated with a lower risk of recurrence in perimenopausal women^17^. Third, tamoxifen and its most active metabolites significantly decreased CRP levels, an estrogenic effect which may contribute to the efficacy of low dose tamoxifen given the association of CRP increase with recurrence shown in previous studies^18,19^.

Endoxifen is below 9 nM in most subjects treated with 5 mg/day and is not predictive of recurrence. Tamoxifen and metabolites were associated with a favorable modulation of IGF-I, CRP, and SHBG levels in peri/postmenopausal women. These liver-mediated, estrogenic effects may contribute to the clinical efficacy of low-dose tamoxifen.

## Methods

### Study design and participants

The study characteristics (EudraCT Number: 2007–007740–10; ClinicalTrials.gov Identifier: NCT01357772, first submitted date: May 17, 2011) and main clinical findings of the trial have previously been reported^2^. Briefly, women aged 75 or younger with ECOG performance status ≤1 and excised hormone-sensitive (estrogen or progesterone receptor ≥1%) or unknown breast intraepithelial neoplasia, including ADH (20%), DCIS (70%), and LCIS (10%), were randomized to either low dose tamoxifen, 5 mg/day, or placebo for 3 years. Women with high-grade or comedo/necrosis DCIS received 50 grays adjuvant radiotherapy. All breast events occurring during the trial were centrally adjudicated by a clinical committee. Treatment compliance was assessed by pill count. The primary endpoint was the incidence of invasive breast cancer or DCIS. Toxicity was assessed by the NCI-CTCAE version 3 and patient-reported menopausal symptoms were recorded by the Breast Cancer Prevention Trial Symptom Scale^2^.

The trial was approved in Italy by the Italian Medicines Agency (AIFA) and by the Ethical Committees for the Coordinating Center (E.O. Ospedali Galliera Ethical Committee) and the Participating Sites. The study was conducted in accordance with the Declaration of Helsinki and guidelines on Good Clinical Practice (ICH E6). All patients were informed of the objectives of the study and were invited to voluntarily participate. Patients who agreed to participate provided written consent before any study-specific procedure that could be withdrawn at any time without consequences for further treatment. Patients received a copy of their rights.

### Laboratory methods

Morning fasting blood samples were collected from a subgroup of patients at baseline, after first and third-year intervention. The samples were stored at −80 °C until assayed. Circulating concentrations of tamoxifen, its metabolites, and biomarkers were determined on serum.

Germline DNA was extracted from whole EDTA-treated blood specimens with QIAamp DNA blood kits (Qiagen, Valencia, CA, USA) according to the manufacturer’s instructions, by the use of the automated platform “Qiacube” (Qiagen, Valencia, CA, USA). DNA was eluted in buffer AE and DNA concentration was quantified using NanoDrop spectrophotometer (Thermo Scientific, Wilmington, DE, USA). DNA concentration was adjusted to 20–70 ng/ul before genotyping. The DNA was amplified in a multiplex PCR reaction and processed for CYP2D6 genotyping by a fully automated multiplex analyzer, the INFINITI™ (AutoGenomics, Carlsbad, CA, USA), according to manufacturer instructions. The technology is based on a detection primer hybridization and extension method applying fluorescent nucleotides. The analysis were performed according to INFINITI CYP450 2DI assay (*2, *2 A, *3, *4, *5, *6, *7, *8, *9, *10, *12, *14, *17, *29, *41 and *XN). According to the predicted enzyme activity the different alleles are categorized in four groups. Full enzyme activity: *1 (wt),*2 (2850 C > T; rs16947), *2 A (−1584 C > G; rs1080985); Null activity: *3 (2549delA; rs35742686), *4 (1846G > A; rs3892097), *5 (CYP2D6 deleted), *6 (1707 delT; rs5030655), *7 (2935 A > C; rs5030867), *8 (1758G > T; rs5030865); *12 (124 G > A; rs5030862); *14 (1758G > A; rs5030865); Reduced activity: and *9 (2615–2617delAGG; rs5030656), *10 (100 C > T; rs1065852), *17 (1023 C > T rs28371706), *29 (1659G > A; rs61736512), and *41 A (2988 G > A; rs28371725); Duplications: *XN: INFINITI does not establishing how many duplicates, nor which specific allele type. If the subject had fully functional alleles in association with a duplication, we assumed the patient was an ultra-rapid metabolizer.

The combination of the two alleles determines the overall genotype effect. Women were classified into four different metabolic phenotypes. Women were defined as extensive metabolizers (EM) if they had 2 fully functional alleles or if they carried one reduced function alleles or one non-functional allele in combination with a fully function allele; intermediate metabolizers (IM) if they carried 2 reduced function alleles or 1 reduced and 1 non-functional allele, while poor metabolizers (PM) carried two non-functional alleles; ultrarapid metabolizers (UM) had fully functional alleles and a duplication.

Serum IGF-I, SHBG, and CRP were measured as previously described^17^. Specifically, serum concentrations of CRP were measured by a high sensitivity latex immunoturbidimetric assay designed for the automated instrument Architect (Abbott Diagnostics, Lake Forest, IL, USA). The lower limit of detection was 0.1 mg/L. Serum SHBG was measured on the same instrument by a chemiluminescent microparticle immunoassay (Abbott Diagnostics, Lake Forest, IL, USA). The lower limit of detection was 0.01 nmol/L. Serum concentrations of IGF-I were determined by a chemoluminescence assay designed for the IDS-isys analyzer (Immunodiagnostic Systems Limited, UK). The method includes an incubation with an acidic solution to dissociate IGF-I from the binding proteins. The lower limit of detection was 4.4 ng/mL.

Serum levels of tamoxifen and 5 metabolites (Z-4OHtam, Z-endoxifen, NDtam, NNDDtam, Tam-NoX) were quantified in samples collected at 1 year (*n* = 169) and 3 years (*n* = 152) using a validated method for serum^5^. All metabolites and four deuterated internal standards (Tamoxifen-d5, 4OHNDtam-d5, Z-4OHtam-d5, NDtam-d5) were obtained commercially. Serum protein precipitation (20 μL) was performed in acetonitrile containing deuterated internal standards using a Hamilton STAR pipetting robot (Bonaduz, Switzerland) and the resulting 80 μL supernatant was evaporated to dryness using nitrogen and reconstituted in 500 μL water:methanol (20:80, v-v). Subsequently, the samples were chromatographically separated on a Waters Aquity UPLC system (Milford, MA, USA) using a Waters BEH Phenyl column (100 mm × 2.1 mm, 1.7 μm particle size) developed by a gradient elution of 0.01% aqueous solution of formic acid and methanol as weak and strong mobile phases, respectively. All gradient steps were linear, and the flow rate was 300 μL/min. The following gradient was used: 0–0.5 min: 95% A and 5% B; 1 min: 65% A and 35% B; 4 min: 10% A and 90% B; 4.5–8 min: 100% B; 8.1–9 min: 90% A and 10% B. Next, the compounds were subjected to atmospheric pressure photoionization and detected in positive ion mode using a Xevo TQ-S tandem mass spectrometer (Waters).

### Statistical analysis

We tested linear relationships between metabolite levels and biomarkers, adjusting for age, BMI, menopausal status, treatment compliance, and baseline biomarker levels. Deviation from normality was graphically checked and log-transformation was performed in case of skewed response variables. The association between biomarker level or metabolite levels and risk of recurrence was tested with Kaplan-Meier survival analysis and Cox proportional hazard model. All statistical tests were two-sided and no correction for multiple testing was adopted.

### Reporting summary

Further information on experimental design is available in the Nature Research Reporting Summary linked to this paper.

## Supplementary information

Supplementary Figure and Material

Data Set 1

Reporting Summary Checklist

## Data Availability

The data generated and analyzed during this study are described in the following data record: 10.6084/m9.figshare.13740826^20^. This is a secondary publication of the trial. The primary publication, which contains a comprehensive description, is 10.1200/JCO.18.01779^2^. The data comprise the following files, in Stata.dta format: ‘Figure1_DeCensiA.dta’, ‘Figure2_DeCensiA.dta’, ‘SeFigure2_DeCensiA.dta’, ‘SeFigure3_DeCensiA.dta’, ‘Table1_DeCensiA.dta’, ‘Results_DeCensiA.dta’. The data are not publicly available for the following reason: to protect patient privacy. However, the data may be made available for collaborative studies upon reasonable request to the corresponding author.

## Code availability

Statistical analyses were performed using STATA (StataCorp. 2015. Stata Statistical Software: Release 14. College Station, TX: StataCorp LP); datasets (“.dta” files) and code (“.do” files) are available upon request for collaborative studies.
