# Supplementary material for: Association of CYP2D6 genotype and tamoxifen metabolites with breast cancer recurrence in a low-dose trial
Source: NPJ Breast Cancer. 2021 Mar 25;7:34. doi: 10.1038/s41523-021-00236-6 (PMC7994552; doi:10.1038/s41523-021-00236-6)
Supplement: Supplementary file 1 — Supplementary Figure and Material [file 41523_2021_236_MOESM1_ESM.pdf]

Supplementary Figure 1: CONSORT Flow Diagram

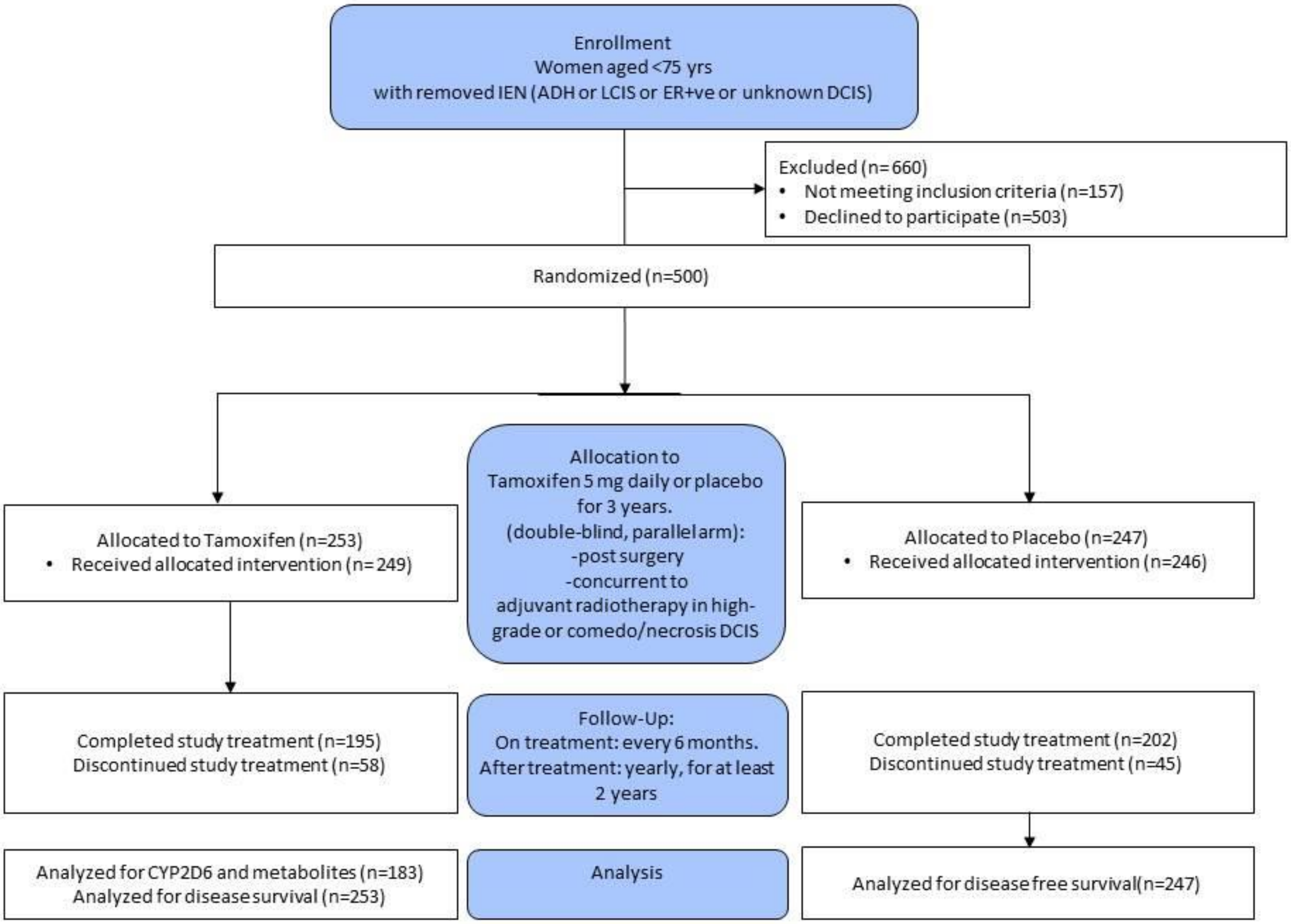

Supplementary Figure 2. Z-endoxifen levels (nmol/L) after 1 year by CYP2D6 polymorphisms assessed by amplichip method.

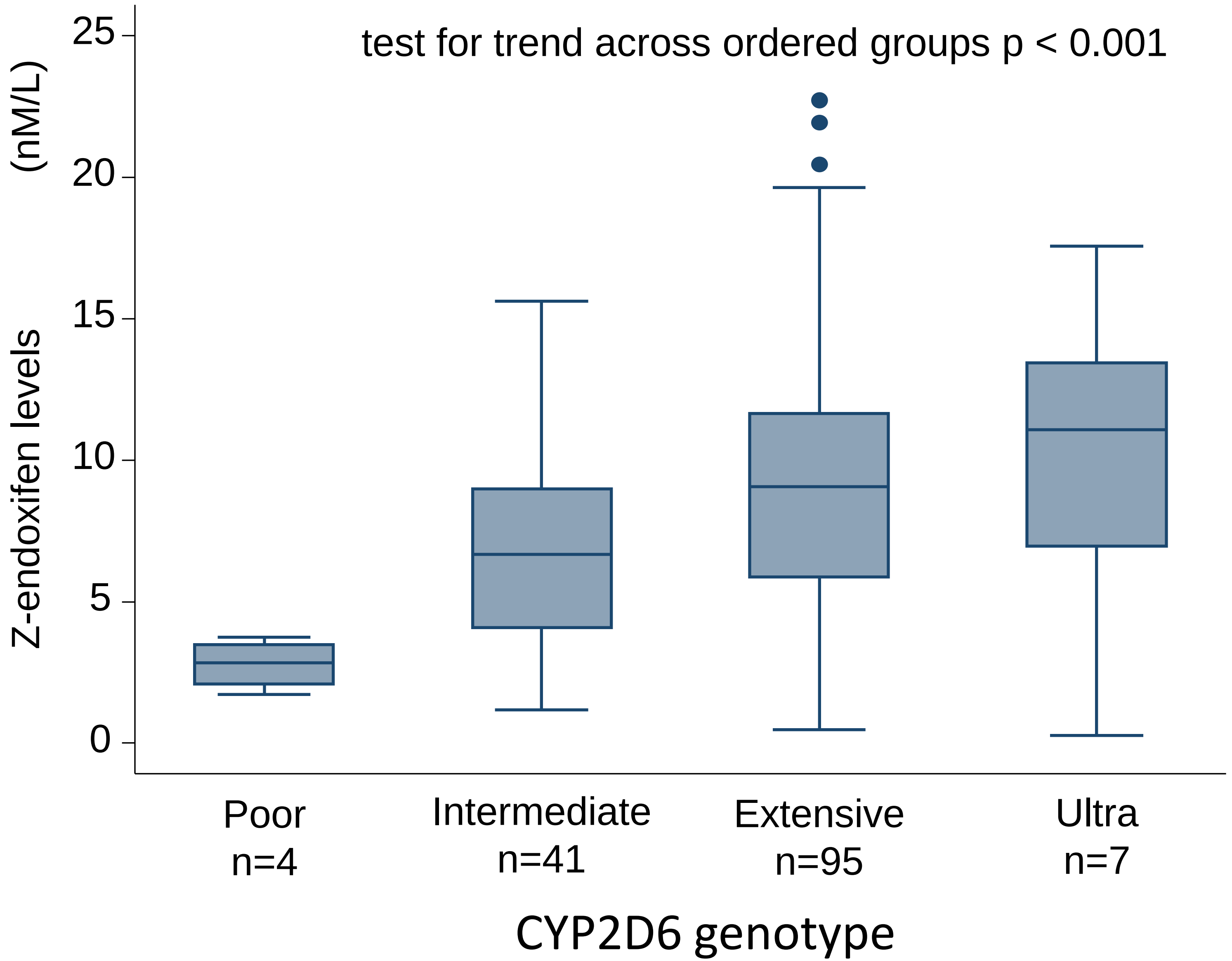

Supplementary Figure 3. Cumulative breast cancer recurrence curves in the tamoxifen arm according to 4OH-tamoxifen, nmol/L and by recurrence status (yes/no).

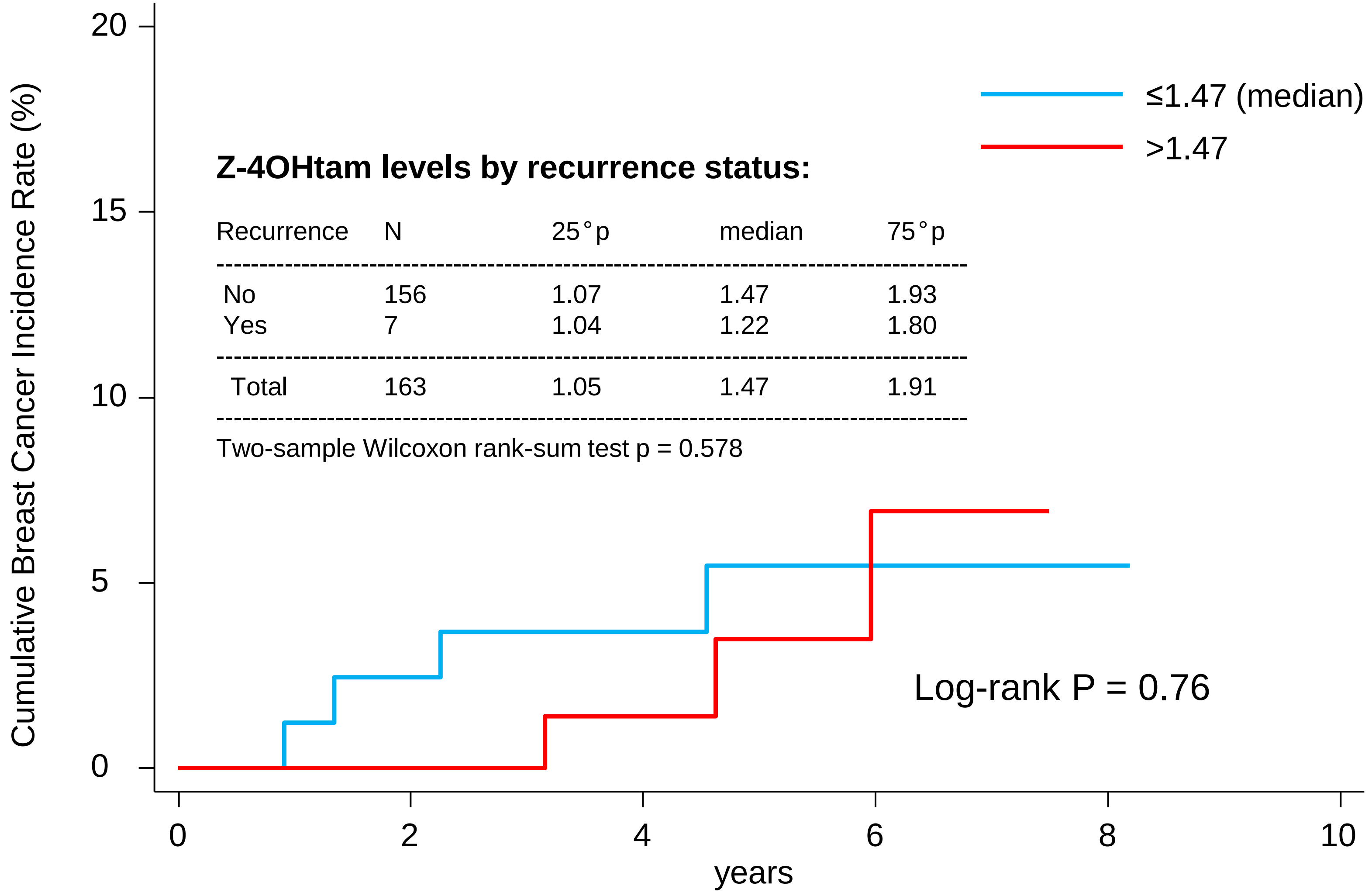

Number at risk

≤1.47	82	(2)	79	(1)	64	(1)	30	(0)	2	(0)	0
>1.47	81	(0)	81	(1)	58	(2)	26	(0)	0	(0)	0

Red line represents subjects with 4OH-tamoxifen level above the median value, the blue line below the median value (log-rank p=0.76).

SUPPLEMENTARY MATERIAL 1

STUDY PROTOCOL

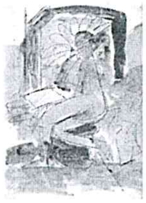

STUDIO RANDOMIZZATO PLACEBO CONTROLLATO DI FASE III CON TAMOXIFEN A BASSE DOSI IN DONNE CON NEOPLASIA INTRAEPITELIALE DELLA MAMMELLA

STUDIO TAM-01

EUDRACT NUMBER: 2007-007740-10

VERSIONE: 5.0 – 20 gennaio 2014

Titolo dello studio/versione/data:	Studio randomizzato placebo controllato di fase III con tamoxifen a basse dosi in donne con neoplasia intraepiteliale della mammella. Studio TAM-01. Versione 5 – 20 gennaio 2014
Fase:	Fase III
Tipologia:	No Profit supportato in parte dal Ministero della Salute (RFPS-2006-1-339898), la Lega Italiana contri I Tumori (LILT 7-08) e l'Associazione Italiana per la Ricerca sul Cancro (AIRC 36/08)
Coordinatore del Progetto:	Andrea De Censi<br>S.C. Oncologia Medica<br>E.O. Ospedali Galliera – Mura delle Cappuccine 14 Genova<br>+39.010.563.4501
Data Management centrale e Monitoraggio:	Sara Campora<br>Ufficio Coordinamento Ricerca Clinica in Oncologia<br>E.O. Ospedali Galliera – Mura delle Cappuccine 14 Genova<br>+39.010.563.4212
Farmacovigilanza:	Marilena Petrera<br>Ufficio Coordinamento Ricerca Clinica in Oncologia<br>E.O. Ospedali Galliera – Mura delle Cappuccine 14 Genova<br>+39.010.563.4580
Analisi Statistica:	Matteo Puntoni<br>Unità di Progettazione e Gestione Sperimentazioni Cliniche – Biostatistica, Ufficio del Coordinatore Scientifico<br>E.O. Ospedali Galliera – Mura delle Cappuccine 14 Genova
Gestione Campioni Biologici e Tissutali:	Harriet Ann Johansson<br>Divisione di Prevenzione e Genetica Oncologica, IEO, Milano<br><br>Giancarlo Pruneri<br>Anatomia patologica e Medicina di Laboratorio, IEO, Milano
Gestione Immagini Mammografiche:	Massimo Calabrese<br>Diagnostica per Immagini e Senologia Diagnostica - IRCCS Azienda Ospedaliera Universitaria San Martino-IST, Genova
Data Monitoring Committee:	Alberto Costa, Paolo Pronzato, Sara Gandini

Accettazione del protocollo di studio/Responsabilità dello Sperimentatore ... 3
1.0 SINOSSI ... 4
2.0 INTRODUZIONE E RAZIONALE ... 6
2.1 Problema clinico/assistenziale ... 7
3.0 OBIETTIVI ... 8
3.1 Obiettivo primario ... 8
3.2 Obiettivi secondari ... 8
4.0 POPOLAZIONE IN STUDIO ... 9
4.1 Criteri di inclusione ... 9
4.2 Criteri di esclusione ... 9
5.0 DISEGNO DELLO STUDIO ... 10
6.0 TRATTAMENTO ... 11
6.1 Nome del trattamento sperimentale ... 11
6.2 Fornitura del farmaco ... 11
6.3 Scelta della dose, gruppi di dosaggio e durata dell'esposizione ... 11
6.4 Formulazione ... 11
6.5 Confezionamento e Etichettatura ... 12
6.6 Somministrazione del trattamento ... 12
6.7 Immagazzinamento ... 12
6.8 Procedure di allocazione ... 12
6.9 Compliance del farmaco ... 13
6.10 Apertura del cieco ... 13
6.11 Tossicità e monitoraggio, modulazione della dose e trattamento degli eventi ... 13
7.0 PROCEDURE DELLO STUDIO ... 15
7.1 Randomizzazione e Scheda Raccolta Dati CRF ... 15
7.2 Visita basale ... 15
7.3 Valutazioni alle visite 6, 12, 18, 24, 30, 36 mesi ... 16
7.4 Farmaci concomitanti ... 16
7.5 Fine Trattamento Sperimentale ... 17
7.6 Piano di follow-up ... 17
7.7 Fine studio ... 17
7.8 Campioni Ematici ... 17
7.9 Campioni Istologici ... 17
7.10 Mammografie ... 18
7.11 Monitoraggi clinici ... 19
8.0 FLOW-CHART ... 19
9.0 EVENTI AVVERSI ... 20
9.1 Definizioni ... 20
9.2 Follow-up ... 20
9.3 Eventi avversi seri (SAE) ... 20
10.0 METODI STATISTICI ... 21
10.1 Dimensione del campione ... 21
10.2 Popolazioni in studio ... 21
10.3 Piano di analisi statistica ... 22
11.0 BIBLIOGRAFIA ... 23

ACCETTAZIONE DEL PROTOCOLLO DI STUDIO/RESPONSABILITÀ DELLO SPERIMENTATORE

Ho letto il protocollo di studio con il seguente titolo:

"STUDIO RANDOMIZZATO PLACEBO CONTROLLATO DI FASE III CON TAMOXIFEN A BASSE DOSI IN DONNE CON NEOPLASIA INTRAEPITELIALE DELLA MAMMELLA" TAM-01

ed accetto di condurre lo studio come specificato nel protocollo e di osservare le norme di Buona Pratica Clinica (GCP), accetto le visite periodiche del monitor e tutte le altre procedure di controllo e verifica dei dati.

Nome dello Sperimentatore Responsabile:

ANDREA DE CENSI

Firma [illegible] Data 20/JAN/2014

1.0 SINOSSI

TITOLO DELLO STUDIO: Studio randomizzato placebo controllato di fase III con tamoxifen a basse dosi in donne con neoplasia intraepiteliale della mammella.

SPERIMENTATORE PRINCIPALE: Andrea De Censi

CENTRO DI COORDINAMENTO: E.O. Ospedali Galliera, S.C.Oncologia Medica, Mura delle Cappuccine, 14 - 16128 Genova, Italia

**Agente In studio**	**Formulazione**	**Dose**	**Regime**	**Via di somm.**
Tamoxifen	5 mg compresse	5 mg/giorno	Ogni giorno	Orale

DISEGNO DELLO STUDIO

Si tratta di uno studio di fase III, controllato, a bracci paralleli, randomizzato, in doppio cieco, con tamoxifen alla dose di 5mg/giorno o placebo per un durata di trattamento di tre anni.

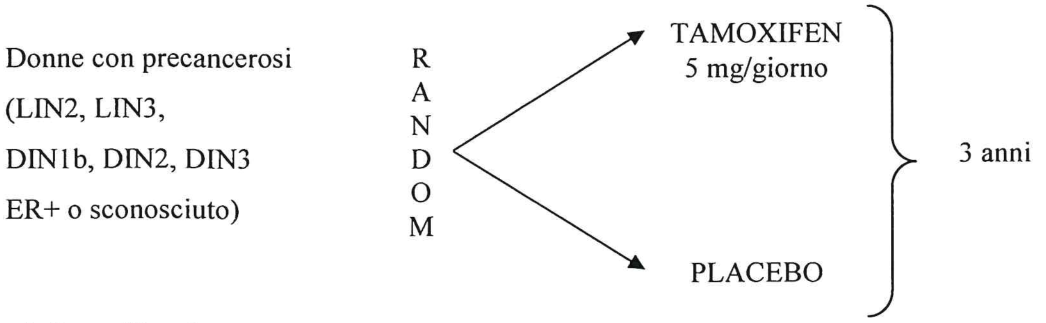

Fattori di stratificazione:
- Centro
- Casi incidenti (diagnosi < 12 mesi) versus casi prevalenti (diagnosi ≥ 12 mesi, < 60 mesi)
- Tipo di lesione (DCIS versus altro)

OBIETTIVI

Primario:
L'obiettivo primario dello studio è valutare se il tamoxifen al dosaggio di 5mg al giorno per 3 anni riduce l'incidenza del cancro mammario invasivo e di carcinoma duttale in situ della mammella, in donne precedentemente operate per neoplasia intraepiteliale lobulare (LCIS ovvero LIN 2 e LIN 3) e duttale (DIN1b, DIN2, DIN3, esclusa DIN 1a) con stato recettoriale ER-positivo o sconosciuto.

Secondari:
Incidenza di altri eventi non invasivi (LCIS, iperplasia atipica), cancro endometriale, ovarico, melanoma, fratture, eventi cardiovascolari, eventi tromboembolici, cataratta clinicamente manifesta, densità mammografica, valutazione livelli circolanti di IGF-I, IGFBP3, SHBG, analisi di polimorfismi genetici dei geni CYP2D6 e CYP2C19 correlati al rischio mammario, analisi centralizzata di ER,PgR Her-2 (se 2+ sottoposti a FISH) e indice di proliferativo dell'antigene Ki-67.

POPOLAZIONE IN STUDIO

Donne di età <75 anni sottoposte ad escissione per neoplasia intraepiteliale lobulare (LIN 2, 3) e duttale (DIN1-3, esclusa DIN 1a), di tipo ER-positivo o sconosciuto. Ogni centro avrà la facoltà di non includere donne con DIN 3 (DCIS di alto grado) se ritiene che il tamoxifen alla dose di 20 mg/die sia il trattamento standard.

CRITERI DI INCLUSIONE

1.Donne con età ≥ 18 e < 75 anni
2.Donne sottoposte ad escissione per neoplasia intraepiteliale lobulare (LCIS, ovvero LIN 2 o 3) e duttale (DCIS, ovvero DIN1b, DIN2 e DIN3, esclusa DIN 1a), di tipo ER-positivo o sconosciuto nei 5 anni (60 mesi) precedenti l'entrata in studio; saranno inclusi sia casi incidenti (diagnosi < 12 mesi) che casi prevalenti (diagnosi ≥ 12 mesi, < 60 mesi), anche ricorrente
3.ECOG Performance status ≤ 1
4.Consenso informato scritto

CRITERI DI ESCLUSIONE

1.Ogni altro tipo di neoplasia invasiva ad esclusione del cancro della pelle non melanomatoso
2.Disordini proliferativi dell'endometrio quali iperplasia atipica, endometriosi, polipi non resecati, mioma sintomatico
3.Alterazioni nella funzionalità epatica, renale e cardiaca di grado ≥ 2 (CTCAE criteria v.3.0)
4.Qualsiasi tipo di disordine retinico, severa cataratta e glaucoma;
5.Presenza di significativi fattori di rischio per eventi venosi, inclusa l'immobilizzazione a seguito di trauma per più di 2 settimane negli ultimi 3 mesi, storia di flebite associata all'uso di estrogeni, tromboflebite venosa profonda o altri significativi eventi trombovenosi (embolia polmonare, infarto, etc.)
6.Precedente uso di tamoxifen, raloxifene o altri SERMs
7.Uso di anastrozolo e altri inibitori dell'aromatasi (IA) negli ultimi 12 mesi per un periodo uguale o superiore ai 6 mesi
8.Terapia anticoagulante in corso con dicumarolo
9.Infezioni attive
10.Severi disordini psichiatrici o incapacità a seguire le procedure del protocollo
11.Inaccessibilità geografica o difficoltà ad assicurare un'adeguata compliance
12.Donne in gravidanza o allattamento
13.Ogni altro fattore che, a discrezione dello sperimentatore, possa controindicare l'uso del tamoxifen

ESAMI DI LABORATORIO

In un sottogruppo di soggetti saranno effettuati prelievi di sangue per l'analisi dei biomarcatori circolanti su campioni di sieri prelevati alla visita basale, alla visita dei 12 mesi e alla visita dei 36 mesi o alla fine del trattamento sperimentale per qualsiasi motivo. Saranno raccolti campioni di sangue intero per le determinazioni dei polimorfismi genetici alla visita basale e alla fine del trattamento sperimentale. Tra i biomarcatori verranno effettuate misurazioni dei livelli circolanti di IGF-I, IGFBP3, SHBG, è prevista l'analisi di alcuni SNPs di geni del citocromo P450 coinvolti nel metabolismo del tamoxifen CYP2D6 (*4,*2, *5, 6* e *7) e CYP2C19. Analisi tissutali verranno condotte centralmente: valutazione dei recettori ormonali (estrogeno e progesterone) ed Her-2, dell'indice proliferativo con la valutazione dell'antigene Ki-67. I casi considerati 2+ per Her-2 (moderata completa immunoreattività di membrana) saranno sottoposti ad esame FISH per conferma. Allestimento di "tissue micro-arrays" con prelievi duplicati da aree dell'inclusione in parafina pre-specificate.

ESAMI STRUMENTALI

Necessaria all'inclusione in studio è una mammografia bilaterale basale seguita da mammografie annuali per tutta la durata dello studio. Se il mammogramma risulterà denso è consigliata l'ecografia mammaria bilaterale. Alla visita basale, e per tutta la durata dello studio, il medico avrà la facoltà di decidere se effettuare l'ecografia transvaginale, comunque consigliata in caso di sintomi.

DURATA DELLO STUDIO: 3 anni di trattamento + 2 anni di follow-up

DATA PRIMA PAZIENTE ARRUOLATA: 12 novembre 2008

DATA STIMATA DI FINE STUDIO: dicembre 2019

2.0 INTRODUZIONE E RAZIONALE

Lo scopo della farmacoprevenzione è l'arresto o la regressione della cancerogenesi prima della fase invasiva con l'uso di sostanze naturali o sintetiche. Recenti studi clinici di fase III dimostrano l'efficacia di tale approccio nel ridurre l'incidenza di cancro mammario, colorettale e prostatico in soggetti a rischio. Tra i determinanti di rischio particolare rilievo riveste la fase dei precursori neoplastici, definita come displasia o neoplasia intraepiteliale, che precede anche di anni il cancro invasivo. Circa il 15-20% delle neoplasie oggi diagnosticate sono in questa fase. Il tumore della mammella è la neoplasia più frequente nella donna. Le donne con neoplasia intraepiteliale operata [iperplasia duttale atipica (ADH), carcinoma lobulare in situ (LCIS) di basso e alto grado (ovvero LIN2 e LIN3) e carcinoma duttale in situ (DCIS) di basso e alto grado, (ovvero DIN1b, DIN2 e DIN3)], con buona prognosi, hanno un rischio annuale di cancro del 10-15/1000 (8-10 volte quello della popolazione generale della stessa fascia di età) e rappresentano un importante target per la farmacoprevenzione.

La FDA (USA) ha approvato l'uso del tamoxifen (TAM), un modulatore selettivo del recettore estrogenico, per la riduzione del rischio di tumore mammario nelle donne ad alto rischio secondo il modello di Gail, che include la presenza di ADH, e nelle donne con pregresso LCIS o DCIS. Nello studio NSABPP1 la riduzione del rischio con TAM 20 mg/d dopo pregressa ADH è stata dell'86% (RR=0.14, 95% IC, 0.03-0.47) e dopo LCIS del 56% (RR=0.44, 95% IC, 0.16-1.06). Per il DCIS il rischio di seconda neoplasia è del 30/1000, e la riduzione del 40% circa.

L'uso del TAM nella pratica clinica è però gravato dalla comparsa di eventi avversi dovuti alla sua parziale attività estrogenica, quali l'aumento del rischio per tumore endometriale e per eventi tromboembolici venosi, che ne limitano l'impiego in farmacoprevenzione. E' stato proposto un nuovo approccio che prevede l'utilizzo di TAM a basse dosi per ridurre il rapporto rischio beneficio. Recenti studi clinici dimostrano che la dose standard di 20 mg/d può essere ridotta a 5 mg/d mantenendo l'attività antiproliferativa sulle cellule tumorali mammarie. Al contrario, il rischio di cancro endometriale è dose-dipendente, e la riduzione del dosaggio può portarne un sostanziale decremento. Inoltre la riduzione di dose è associata a una globale diminuzione dell'attività estrogenica del farmaco su IGF-I, SHBG e antitrombina-III, cui consegue una riduzione del rischio di eventi trombotici. Infine il tamoxifene ha un'elevata distribuzione tissutale ed è dimostrato che la dose di 5 mg/d garantisce a livello del tessuto mammario una concentrazione circa 10 volte superiore a quella che in vitro è in grado di inibire la crescita cellulare.

E' dimostrato che il trattamento della displasia o precancerosi porta ad una riduzione dell'insorgenza di neoplasie invasive. L'attuale carenza di protocolli clinici standardizzati per il trattamento delle precancerosi sottolinea la grande importanza e la necessità di realizzare un trial clinico di farmacoprevenzione che possa validare l'utilizzo del tamoxifen a basse dosi nelle donne portatrici di tali patologie ad elevato rischio evolutivo.

La dimostrazione dell'efficacia e della sicurezza di un simile trattamento nel prevenire le neoplasie mammarie invasive comporterebbe non solo un notevole guadagno in termini di sopravvivenza e di qualità della vita per tutte le pazienti ad aumentato rischio, ma anche, in termini di implicazioni per il SSN, una contrazione della spesa sanitaria in ragione della riduzione dell'impiego di interventi costosi (in termini economici e umani) e del basso costo del tamoxifen (circa 10 euro/mese), farmaco non più coperto da brevetto.

2.1 Problema clinico/assistenziale

Attualmente non esiste uno standard per il trattamento delle lesioni mammarie displastiche o preinvasive. Ciò si riflette in un'ampia eterogeneità di atteggiamenti terapeutici, che variano dalla semplice sorveglianza clinico-strumentale periodica dopo escissione chirurgica della lesione all'impiego di trattamenti aggressivi, quali la mastectomia o la radioterapia, o all'impiego di farmaci antitumorali di nuova generazione, quali gli inibitori dell'aromatasi, attivi nel trattamento delle neoplasie invasive ma ancora non registrati per la farmacoprevenzione a causa della mancanza di dati di efficacia e sicurezza a lungo termine derivanti da trials clinici controllati.

E' implicita l'importante ricaduta economica che tali strategie hanno sul SSN: alti costi per esami strumentali ripetuti, per degenze più o meno protratte in strutture chirurgiche, per l'impiego di metodiche o tecnologie ad alta specializzazione e, infine, per l'impiego di farmaci costosi sempre più oggetto di spinte commerciali. La standardizzazione di un trattamento che si dimostri in grado di prevenire la comparsa di neoplasie mammarie in donne ad alto rischio mediante l'impiego di un farmaco efficace, sicuro e a basso costo rappresenterebbe pertanto un notevole passo avanti, in termini sia umani che economici.

3.0 OBIETTIVI

3.1 Obiettivo primario

L'obiettivo primario dello studio è valutare se il tamoxifen al dosaggio di 5mg al giorno per 3 anni riduce l'incidenza del cancro mammario invasivo e di carcinoma duttale in situ della mammella (DIN 2 e DIN 3).

3.2 Obiettivi secondari

- Incidenza di altri eventi non invasivi (i.e., LCIS, iperplasia atipica)
- Valutazione della tollerabilità del tamoxifen: incidenza di carcinoma endometriale, ovarico, eventi tromboembolici
- Incidenza di fratture, eventi cardiovascolari e cardiaci arteriosi, cataratta clinicamente manifesta e melanoma
- Valutazione della densità mammografica
- Analisi centralizzata in un sottogruppo di soggetti delle misurazioni dei livelli circolanti di IGF-I, IGFBP-3, SHBG. E' prevista l'analisi di alcuni SNPs dei geni del citocromo P450 coinvolti nel metabolismo del tamoxifen CYP2D6 (*4, *2, *5, *6 e *7), CYP2C19
- Analisi centralizzata dell'indice proliferativo con la valutazione dell'antigene Ki-67, ER, PgR, HER2 con immunoistochimica (IHC) sui preparati istologici; allestimento di "tissue micro-arrays" con prelievi duplicati da aree dell'inclusione sui preparati istologici. Estrazione del DNA/RNA e valutazione dell'impronta" genetica PAM50 come valore prognostico ed end-point surrogato di rischio di cancro mammario

4.0 POPOLAZIONE IN STUDIO

Si tratta di donne di età inferiore ai 75 anni sottoposte ad escissione per neoplasia intraepiteliale lobulare (LIN 2, 3) e duttale (DIN1b, DIN2 e DIN3, esclusa DIN 1a), di tipo ER-positivo o sconosciuto. Verranno inclusi, previa stratificazione, sia casi incidenti (diagnosi < 12 mesi) che prevalenti (diagnosi ≥ 12 mesi, < 60 mesi). La determinazione dell'espressione dei recettori ormonali è ormai parte della pratica clinica per i DCIS, mentre per ADH e LCIS non è di routine essendo la percentuale di positività molto elevata. Ogni centro avrà la facoltà di non includere donne con DIN 3 (DCIS di alto grado) se ritiene che il tamoxifen alla dose di 20 mg/die sia il trattamento standard.

4.1 Criteri di inclusione

1. Donne con età ≥ 18 e < 75 anni
2. Donne sottoposte ad escissione per neoplasia intraepiteliale lobulare (LCIS, ovvero LIN 2 o 3) e duttale (DCIS, ovvero DIN1b, DIN2 e DIN3, esclusa DIN 1a), di tipo ER-positivo o sconosciuto nei 5 anni (60 mesi) precedenti l'entrata in studio; saranno inclusi sia casi incidenti (diagnosi < 12 mesi) che casi prevalenti (diagnosi ≥ 12 mesi, < 60 mesi), anche ricorrente
3. ECOG Performance status ≤ 1
4. Consenso informato scritto

4.2 Criteri di esclusione

1. Ogni altro tipo di neoplasia invasiva ad esclusione del cancro della pelle non melanomatoso
2. Disordini proliferativi dell'endometrio quali iperplasia atipica, endometriosi, polipi non resecati, mioma sintomatico
3. Alterazioni nella funzionalità epatica, renale e cardiaca di grado ≥ 2 (CTCAE criteria v.3.0)
4. Qualsiasi tipo di disordine retinico, severa cataratta e glaucoma
5. Presenza di significativi fattori di rischio per eventi venosi, inclusa l'immobilizzazione a seguito di trauma per più di 2 settimane negli ultimi 3 mesi, storia di flebite associata all'uso di estrogeni, tromboflebite venosa profonda o altri significativi eventi trombovenosi (embolia polmonare, infarto, etc.)
6. Precedente uso di tamoxifen, raloxifene o altri SERMs
7. Uso di anastrozolo e altri inibitori dell'aromatasi (IA) negli ultimi 12 mesi per un periodo uguale o superiore ai 6 mesi
8. Terapia anticoagulante in corso con dicumarolo
9. Infezioni attive
10. Severi disordini psichiatrici o incapacità a seguire le procedure del protocollo
11. Inaccessibilità geografica o difficoltà ad assicurare un'adeguata compliance
12. Donne in gravidanza o allattamento
13. Ogni altro fattore che, a discrezione dello sperimentatore, possa controindicare l'uso del tamoxifen

5.0 DISEGNO DELLO STUDIO

Studio di fase III, controllato, a bracci paralleli, randomizzato, in doppio cieco, dotato del seguente schema di trattamento:

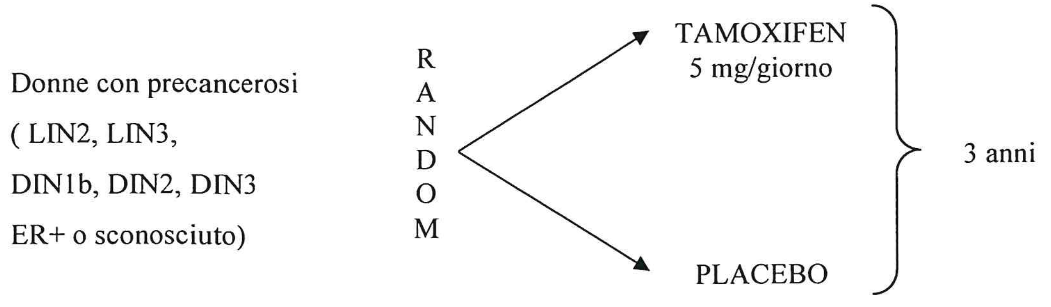

Fattori di stratificazione:

- Centro partecipante
- Casi incidenti (diagnosi < 12 mesi) versus casi prevalenti (diagnosi ≥ 12 mesi, < 60 mesi)
- Tipo di lesione (LIN versus DIN)

Si tratta di un trial multicentrico, nazionale, che prevede un arruolamento di 5 anni. Il trattamento è previsto per 3 anni con 2 anni aggiuntivi di follow-up.

6.0 TRATTAMENTO

Tutte le partecipanti assumeranno per 3 anni per via orale una compressa al giorno di tamoxifen 5 mg o placebo.

6.1 Nome del trattamento sperimentale

Nome Generico: **tamoxifen**

Nome chimico: (z)-2-[4-(1,2-Dyphenyl-1-butenyl)phenoxy]-N,N-dymethylethanamine citrate

Classificazione: antagonista estrogenico non steroideo

CAS Registry No: 10540-29-1

Formula molecolare: C26H29NO.C6H8O7

Massa molecolare: 563.65

Principali caratteristiche chimiche: fine polvere bianca cristallina inodore, punto di fusione 144°C, leggermente solubile in acqua, solubile in metanolo, cloroformio, acetone, igroscopica ad alte umidità relative.

6.2 Fornitura del farmaco

La fornitura del farmaco (tamoxifen 5mg) e del placebo confezionato secondo le normative vigenti (GMP) è totalmente a carico del centro coordinatore E.O.Ospedali Galliera di Genova e sarà fornito ai centri partecipanti pronto per essere consegnato alle pazienti randomizzate nello studio.

6.3 Scelta della dose, gruppi di dosaggio e durata dell'esposizione

La scelta di una dose giornaliera di 5 mg di tamoxifen si basa su studi precedenti di incremento della dose; tali studi indicano un'attività della dose di 5mg/die praticamente identica rispetto alla dose standard utilizzata di 20mg/die nella modulazione di un set di biomarcatori surrogati. Inoltre, l'attività antiproliferativa del tamoxifen a dosi differenti è stata recentemente determinate in uno studio pre-operatorio in doppio cieco di 4 settimane su un numero di 120 donne affette da cancro mammario. I primi risultati indicano riduzioni simili del ki67 rispetto al basale, ottenuto da una biopsia, sia nella somministrazione di tamoxifen a dose di 5mg che in quella a 20mg.
I soggetti assumeranno tamoxifen o placebo per un periodo complessivo di 3 anni. Le compresse di tamoxifen da 5mg e il placebo saranno confezionate in flaconi. L'assunzione del farmaco o del placebo sarà nell'ordine di una compressa al giorno. Alle pazienti verrà consegnato un flacone contenente farmaco sufficiente per un periodo di somministrazione di sette mesi (210 compresse), il quale dovrà essere restituito agli investigatori alla successiva visita semestrale.

6.4 Formulazione

Compresse di tamoxifen: citrato di tamoxifen, cellulosa microcristallina, amido di mais, povidone, stearato di magnesio, sodio croscarmelloso, idrossipropil metil-cellulosa, polietilene glicole 300, diossido di titanio.
Compresse di placebo: cellulosa microcristallina, amido di mais, povidone, stearato di magnesio, sodio croscarmelloso, idrossipropil metil-cellulosa, polietilene glicole 300, diossido di titanio.

6.5 Confezionamento ed Etichettatura

Il Tamoxifen verrà fornito in flaconi da 210 compresse di composto attivo. La preparazione risulterà visivamente identica a quella del placebo.

Il trattamento sarà assegnato dall'Ufficio Coordinamento Ricerca Clinica in Oncologia dell'Ospedale Galliera. L'assegnazione avverrà in doppio cieco, ossia né gli sperimentatori coinvolti nello studio, né le donne partecipanti sapranno quale tipo di compressa (tamoxifen o placebo) sarà somministrata. Il tipo di trattamento e il relativo braccio di appartenenza del paziente saranno resi noti dal centro coordinatore, previa formale richiesta scritta dello sperimentatore principale, solamente in caso di comprovata necessità (eventi avversi seri) accertata dal responsabile scientifico dello studio.

Le informazioni annotate su tutte le etichette del farmaco sperimentale (tamoxifen o placebo) sono: "Studio TAM-01 - Periodo di 6 mesi: - Da utilizzarsi solamente per studi sperimentali – Codice numero – Numero di lotto – Data di re-test - Indirizzo e numero di telefono per urgenze – Formula (tamoxifen 5mg o placebo) - Dosaggio (1 compressa/die uso orale) – Avvertenze (Tenere fuori dalla portata dei bambini)".

Una porzione dell'etichetta risulterà removibile e verrà applicata su un apposito form al fine di assicurare un ulteriore controllo sulla corretta assegnazione del farmaco.

6.6 Somministrazione del trattamento

Le pazienti arruolate nello studio si autosomministreranno il farmaco, una compressa di tamoxifen/placebo al giorno, per un periodo complessivo di 3 anni.

6.7 Immagazzinamento

I farmaci dello studio verranno conservati presso la S.C. Farmacia dell'E.O. Ospedali Galliera e conservati secondo normativa vigente. Tutti i centri partecipanti al trial dovranno attenersi alle norme di GCP per la conservazione dei farmaci sperimentali in loco. Il giorno della randomizzazione il farmaco sarà consegnato alle pazienti e il personale addetto dovrà registrare e siglare questa procedura su un form di contabilità fornito dal centro coordinatore.

6.8 Procedure di allocazione

La randomizzazione avverrà via web attraverso un sistema informatico automatizzato generato dal Centro per il Coordinamento delle Sperimentazioni Cliniche presso l'Istituto Toscano Tumori di Firenze e congiuntamente gestito con l'Ufficio Coordinamento Ricerca Clinica in Oncologia dell'Ospedale Galliera.

Il numero di trattamento sarà generato automaticamente dal sisitema producendo un identificativo del paziente, corrispondente al contenuto del farmaco (tamoxifen o placebo), che sarà presente sull'etichetta del flacone.

Tutto il personale coinvolto nello studio lavorerà in cieco rispetto all'allocazione del trattamento fino allo smascheramento dello stesso (vedi paragrafo 6.10).

6.9 Compliance del farmaco

Per gestire la contabilità del farmaco sperimentale verrà redatto da ogni centro e per ogni paziente un apposito modulo di contabilità fornito dal centro coordinatore al momento dell'apertura del centro.

Ogni centro autonomamente si farà carico dello smaltimento del farmaco residuo. Qualora un centro non possa smaltire in autonomia il farmaco residuo, su richiesta scritta e motivata, il centro coordinatore valuterà la possibilità di raccogliere e distruggere il farmaco presso il centro di coordinamento.

6.10 Apertura del cieco

L'apertura del doppio cieco durante la fase attiva del trattamento in studio verrà accordata, a tutela della salute del paziente, solo in casi di assoluta necessità e sotto esplicita e motivata richiesta del medico sperimentatore.

Qualora sussista l'urgente necessità di apertura del cieco, per gravi condizioni cliniche del soggetto o per ogni altra condizione che garantisca la tutela della salute del soggetto stesso, lo sperimentatore responsabile del centro potrà rompere il cieco in autonomia, comunicando solo successivamente l'accaduto.

Gli eventi che potranno condurre all'apertura del cieco sono i seguenti:

1. tossicità del farmaco di grado 3 o superiore
2. qualsiasi altro tipo di evento avverso grave, inclusi cancro alla mammella o in altri siti
3. completamento dello studio o specifica richiesta del data center

6.11 Tossicità e monitoraggio, modulazione della dose e trattamento degli eventi

La tossicità verrà valutata ad ogni visita in base ai criteri di tossicità CTCAE (versione 3.0, http://www.fda.gov/cder/cancer/toxicityframe.htm).

Gli eventi avversi più conosciuti del tamoxifen si manifestano attraverso i seguenti sintomi:

- endocrini (vampate di calore)
- fisici (sudorazione, aumento di peso)
- sessuali e riproduttivi (perdite vaginali, secchezza e prurito vaginale, sanguinamenti, irregolarità del ciclo mestruale, dispareunia)
- genitourinari (incontinenza)

Sarà normalmente richiesto alle pazienti di mantenere la dose piena per tutta la durata del trattamento sperimentale. Tuttavia, nel caso si manifestino delle tossicità, verrà applicata una modifica della dose che sarà accuratamente registrata.

In caso di tossicità di grado 1, la paziente continuerà comunque il trattamento a dose completa. La tossicità verrà in seguito controllata in base alla sua rilevanza clinica.

In caso di tossicità di grado 2 o superiore la dose sarà modificata in accordo al potenziale grado di correlazione con il trattamento sperimentale come indicato nella seguente tabella:

Tossicità	Potenziale grado di correlazione al trattamento				
**Grado**	**Non correlato**	**Poco probabile**	**Possibile**	**Probabile**	**Correlato**
1	C	C	C	C	C
2	C	C	S-R[0]	S-R[1]	S-R[1]
3	S-R[2]	S-R[2]	S-R[1]	W	W

**4**	S-R^2	S-R^1	W	W	W

C = il trattamento continua a dose piena.

S-R^0 = interruzione del trattamento fino all'abbassamento della tossicità a grado 1 o inferiore e ripresa del trattamento a dose piena. Se la tossicità di grado 2 ricomparirà, il trattamento verrà interrotto nuovamente e, al raggiungimento della tossicità di grado 1 o inferiore, verrà ripreso ad un dosaggio ridotto del 50% (1 compressa ogni 2 giorni) e tale rimarrà fino alla fine dello studio.

S-R^1 = interruzione del trattamento fino all'abbassamento della tossicità grado 1 o inferiore, e ripresa al dosaggio ridotto del 50% (1 compressa ogni 2 giorni) fino alla fine dello studio.

S-R^2 = interruzione del trattamento fino all'abbassamento della tossicità grado 1 o inferiore, e ripresa al dosaggio ridotto del 50% (1 compressa ogni 2 giorni) per un mese. Se la tossicità non supererà il grado 1, sarà ripreso il trattamento a dose piena. In caso contrario (i.e. ricomparsa tossicità > grado 1), il trattamento verrà nuovamente interrotto fino all'abbassamento della tossicità a grado ≤ 1, a quel punto verrà nuovamente ridotta la dose del 50% (1 compressa ogni 2 giorni) e tale sarà mantenuta. Se la tossicità non si risolve dopo la riduzione di dose, un'ulteriore riduzione del dosaggio del 50% (1 compressa ogni 4 giorni) verrà utilizzata fino alla fine dello studio. Dopo tale seconda riduzione non sarà possibile effettuare ulteriori riduzioni di dose e il paziente che manifesterà ulteriori tossicità verrà rimosso dallo studio (W).

W = Il trattamento verrà interrotto e il paziente uscirà dallo studio.

7.0 PROCEDURE DELLO STUDIO

I dati originali della sperimentazione clinica devono essere conservati e registrati in una cartella clinica ospedaliera; qualora tali cartelle per soggetti ambulatoriali non siano previste dalla prassi della struttura o dell'ambulatorio sede della sperimentazione, le stesse dovranno essere predisposte ai fini della normativa vigente (GCP). Nel caso vengano utilizzate le Schede Raccolta Dati cartacee come "source document" è necessario che lo sperimentatore principale del centro dichiari le modalità di archiviazione di tali documenti e dopo averli firmati, timbrati e datati li inserisca nella cartella ambulatoriale come parte integrante della stessa.
Tutti i documenti della sperimentazione dovranno essere conservati presso il centro arruolante per 15 anni dal termine della sperimentazione.

7.1 Randomizzazione e Scheda Raccolta Dati CRF

Dopo aver ottenuto il consenso informato le pazienti eleggibili possono essere randomizzate.
Per l'assegnazione al gruppo di trattamento sarà utilizzato un un sistema informatico validato, attivo 24 ore al giorno, al seguente inidirizzo https://www.eclintrials.org. Ad ogni sperimentatore e personale addetto alla randomizzazione sarà assegnato un nome utente personale (quando il centro coordinatore ha ottenuto i documenti amministrativi di abilitazione del centro) ed una password personale. Al termine del processo di randomizzazione, la pagina informatizzata potrà essere stampata e inserita nel dossier del paziente e rappresenterà la notifica di randomizzazione. A questo punto sarà abilitata la funzione di inserimento dati nei moduli eCRF. Secondo un piano di validazione dei dati, saranno effettuati controlli di inserimento e qualità e saranno generate dal sistema informatico forme di chiarimento (DCF), in visibilità agli sperimentatori o al personale addetto di ogni centro, sul sito stesso. Le DCF dovranno essere risolte da ogni centro, on-line e il cambiamento/inserimento/congruità dei dati corretti nella eCRF sarà effettuata dal centro coordinatore.

7.2 Visita basale

Durante la visita basale sarà valutata la mammografia e l'ecografia transvaginale, se eseguita, sarà effettuato il primo prelievo per i biomarkers ed è consigliabile effettuare anche il prelievo per la farmacogenetica. Sarà somministrato alla paziente il questionario sui sintomi vasomotori, una volta verificata l'eligibilità della paziente sarà randomizzata e le sarà consegnato il farmaco. Saranno raccolti i seguenti dati: data di nascita, età al menarca, n. figli, età al primo parto, stato menopausale, data ultima mestruazione, età alla menopausa, familiarità 1°e 2° grado per carcinoma della mammella e per carcinoma dell'ovaio, utilizzo di terapia ormonale, uso di alcool e fumo, patologie precedenti attuali, peso, altezza, circonferenza pubica e ombelicale. Sarà effettuato un esame obiettivo e sarà segnata la data di consegna del farmaco sperimentale specificando il codice del trattamento assegnato, il numero di compresse totali consegnate, l'eventuale assunzione di farmaci concomitanti.
Saranno raccolti in questa visita i dati sulla neoplasia intraepiteliale, nello specifico:
data intervento chirurgico, sede intervento, tipo di intervento, biopsia linfonodo sentinella, numero eventuali precedenti interventi/biopsie/ago aspirati per condizione benigna della mammella. Dati anatomo patologici: data

diagnosi istologica, istotipo, presenza di comedo, presenza necrosi, stadio pT,pN, numero linfonodi rimossi, numero linfonodi positivi, tipo neoplasia, neoplasia multicentrica, dimensioni del nodulo maggiore. Localizzazione. Marcatori biologici: recettori progestinici, recettori estrogenici, HER2/neu, espressione KI-67.
I sintomi vasomotori associati all'uso del tamoxifen saranno valutati come descritto da Stanton, Bernaards, e Ganz e per quanto riguarda numero e intensità di vampate di calore, secondo quanto descritto da Sloan A, Loprinzi CL et al.

7.3 Valutazioni alle visite 6, 12, 18, 24, 30, 36 mesi

Un'intervista medica, una visita clinica e una valutazione dei possibili sintomi ed eventuali eventi avversi gravi saranno ripetute ogni 6 mesi (± 1 mese). E' obbligatoria una visita annuale al seno, le visite del 6°, 18° e 30° mese potranno essere effettuate da personale di ricerca clinica o infermieristico addestrato sotto la responsabilità del medico.
La mammografia verrà ripetuta ogni 12 mesi per valutare l'effetto dell'intervento al seno, ed in caso di mammogramma denso l'ecografia è raccomandata.
Un esame ginecologico e l'ecografia transvaginale saranno eseguiti se indicati e ripetuti in caso di sintomi, quali ad esempio il sanguinamento imprevisto.
I prelievi per i biomarcatori circolanti saranno eseguiti solo alle visite dei 12 e 36 mesi o alla fine del trattamento in caso di sospensione per altro motivo. Sarà somministrato alla paziente il questionario sui sintomi vasomotori, sarà ritirato il farmaco residuo o il flacone vuoto, sarà verificata la compliance al trattamento anche attraverso il diario auto compilato dalle pazienti e sarà consegnato il nuovo flacone, ad esclusione dell'ultima visita (36 mesi).
Saranno raccolti i seguenti dati: data della visita, peso, circonferenza pubica e ombelicale, esame obiettivo, ecografia transvaginale (eseguita si/no, data esecuzione, alterata si/no, spessore rima endometriale); mammografia (eseguita si/no, data esecuzione, alterata si/no); radioterapia (eseguita si/no, data inizio/fine, dose totale); compliance al trattamento (numero compresse consegnate all'ultima visita, numero compresse restituite alla visita attuale, numero compresse consegnate alla visita attuale, data visita attuale) applicando l'etichetta del flacone consegnato sull'apposito modulo cartaceo; terapie concomitanti da rilevare sull'apposito modulo cartaceo; valutazione degli eventi avversi secondo CTCAE v3.0 sono da rilevare sull'apposita sezione delle CRF.

7.4 Farmaci concomitanti

I trattamenti farmacologici concomitanti saranno verificati a ogni visita e riportati nell'apposito form cartaceo fornito dal centro coordinatore. L'uso di farmaci anticoagulanti dicumarolici e dei seguenti farmaci inibitori del citocromo CYP2D6, interferiscono con il metabolismo del tamoxifen: *paroxetina, fluxetina, sertralina, fluvoxamina, venlafaxina, clomipramina, amitriptilina, cimetinida, flufenazina, aloperidolo, perfenazina, tioridazina.* **La loro prescrizione è sconsigliata** e comunque il loro utilizzo deve essere giustificato e accuratamente registrato nel form cartaceo sopradescritto.

7.5 Fine Trattamento Sperimentale

Il trattamento sperimentale si considera concluso quando le pazienti terminano i 3 anni di auto somministrazione del farmaco. La pazienti che non completeranno il periodo di trattamento verranno classificate come uscite anticipatamente dallo studio. Alcuni possibili motivi per l'uscita anticipata dallo studio includono: un arruolamento inappropriato; violazioni di protocollo; diagnosi di neoplasia mammaria post-basale; evento avverso; rifiuto della paziente a continuare lo studio; paziente persa al follow-up; decesso.

7.6 Piano di follow-up

Al termine del trattamento sperimentale le pazienti saranno seguite con una visita annuale o un contatto telefonico per ulteriori due anni. Durante la visita/telefonata saranno rilevate eventuali sintomatologie legate all'assunzione del farmaco e/o un'eventuale insorgenza di neoplasia mammaria.
Per le pazienti che ritirano il consenso informato non saranno raccolti i dati.

7.7 Fine studio

L'arruolamento terminerà a Dicembre 2014 e pertanto lo studio si considererà terminato quando l'ultima paziente arruolata avrà concluso i 3 anni di trattamento e i 2 anni di follow-up.

7.8 Campioni Ematici

In un sottogruppo di soggetti saranno raccolti campioni di sangue alla visita basale e dopo 12 e 36 mesi di trattamento. Sarà istituita una biobanca per l'analisi dei biomarcatori circolanti su campioni di siero che saranno raccolti, in caso di interruzione del trattamento sperimentale per qualsiasi motivo. Inoltre saranno raccolti campioni di sangue intero per le determinazioni dei polimorfismi genetici (DNA genomico) alla visita basale, alla conclusione del trattamento (per qualsiasi motivo) e a conclusione dello studio dopo i due anni di follow up. La biobanca sarà centralizzata presso l'Istituto Europeo di Oncologia – Divisione di Prevenzione e Genetica Oncologica – che si occuperà di fornire ai centri arruolanti dei kits per lo stoccaggio dei campioni con etichette a codice a barre, nonché di coordinare le spedizioni dai centri all'IEO e verificare la corretta identificazione dei campioni presso la biobanca centralizzata. Tra i biomarcatori verranno effettuate misurazioni dei livelli circolanti di IGF-I, IGFBP-3, e SHBG, ed è prevista l'analisi di alcuni SNPs di geni del citocromo P450 coinvolti nel metabolismo del tamoxifen CYP2D6 (*4, *2, *5, *6 e *7) e CYP2C19. La biobanca fornirà l'analisi tecnica e scientifica e l'interpretazione dei dati. Una guida tecnica dettagliata sarà fornita a i centri arruolanti per la gestione dei prelievi.

7.9 Campioni Istologici

Inclusioni in paraffina del materiale inerente l'intervento chirurgico saranno raccolte dai centri arruolanti e inviate presso la Divisione di Anatomia Patologica dell'Istituto Europeo di Oncologia, recentemente

accreditata Joint Commission ed attualmente coinvolta come centro revisore in numerosi studi clinici internazionali di fase III sul carcinoma mammario. Ogni inclusione verrà utilizzata per ottenere un preparato in ematossilina ed eosina per la conferma istologica, e per la valutazione dei recettori ormonali (estrogeno e progesterone) ed Her-2, con sonde approvate dalla Food and Drug Administration (FDA), e dell'indice proliferativo con la valutazione dell'antigene Ki-67. La valutazione di ER, PgR ed Her-2 verrà condotta seguendo le raccomandazioni dell'ASCO/CAP (cut-offs: > o = 1% di cellule neoplastiche per ER e PgR e > o = 10% di cellule con intensa e completa immunoreattività di membrana). I casi considerati 2+ per Her-2 (moderata completa immunoreattività di membrana) saranno sottoposti ad esame FISH per conferma con sonde approvate dall'FDA. Al fine di consentire successivi studi traslazionali, l'Unità provvederà inoltre ad allestire "tissue micro-arrays" con prelievi duplicati da aree dell'inclusione pre-specificate sui preparati istologici.

7.10 Mammografie

Casi incidenti (diagnosi < 12 mesi): la mammografia va effettuata prima dell'ingresso in studio. Se la paziente viene randomizzata entro i 6 mesi dopo l'intervento chirurgico ed ha effettuato la mammografia prima dell'intervento (si tratta quindi di mammografia con malattia evidente) questa non dovrà essere ripetuta per l'inclusione in studio.

Casi prevalenti (diagnosi >12 mesi, < 60 mesi): la mammografia va effettuata prima dell'ingresso in studio se la mammografia precedente è più vecchia di 1 anno.

In entrambi i casi: ogni centro dovrà fornire anche la mammografia della diagnosi pre-intervento (esempio: caso prevalente con mammografia dell'01/01/2009 e data intervento il 10/01/2009. Randomizzazione l'01/06/2011 con mammografia datata 01/05/2011→ il centro arruolante dovrà fornire la mammografia pre-intervento dell'01/01/2009 e la mammografia dell'inclusione in studio del 01/05/2011).

Mammografia follow-up: le mammografie di controllo andranno eseguite ogni 12 mesi a partire dalla mammografia basale quindi indipendentemente dalle visite di controllo.

Le mammografie dovranno essere eseguite secondo le seguenti indicazioni tecniche:

- mammografia bilaterale digitale o analogica digitalizzata,
- registrazione su supporto rigido CD in formato dicom, con anonimizzazione dei dati personali della paziente.

Dovrà essere riportata la data di effettuazione dell'esame e il codice di trattamento della paziente (n.centro.n.confezione, esempio 01.09, 03.15).

Qualora i centri arruolanti fossero impossibilitati a rendere anonime le immagini, il centro di raccolta provvederà all'anonimizzazione in fase di ricezione e di revisione dei dischetti.

Nel caso il centro arruolante possieda solo le lastre della paziente queste andranno inviate al centro di raccolta che provvederà alla digitalizzazione ed alla restituzione delle stesse dopo 30 giorni lavorativi dal ricevimento. La raccolta delle immagini sarà gestita dal centro coordinatore che provvederà ad inviarle c/o Azienda Sanitaria San Martino di Genova per la valutazione ed analisi dei dati. Una guida tecnica dettagliata sarà fornita ai centri arruolanti per la gestione raccolta e invio delle immagini.

7.11 Monitoraggi clinici

Saranno effettuati dei monitoraggi clinici da personale qualificato presso i centri arruolanti sulla base di:

- difficoltà arruolamento
- arruolamento molto attivo
- problematiche tecnico-organizzative

In occasione dei monitoraggi, l'investigatore principale del centro dovrà mettere a disposizione tutti i documenti dello studio, le cartelle cliniche dei soggetti, il farmaco e tutto il materiale inerente il trial.

8.0 FLOW-CHART

Valutazione/Procedure	Basale	Ogni 6 mesi (± 1 mese)	Ogni 12 mesi (± 1 mese)	Drop Out	Follow-up (annuale)
Consenso informato	X				
Eleggibilità	X				
Storia medica	X				
Stile di vita	X				
Terapie concomitanti	X	X		X	X
Randomizzazione	X				
Somministrazione farmaco	X	X			
Esame clinico	X	X(1)		X	(X)
Visita senologica	X		X	X	(X)
Mammografia	X(2)		X(2)	(X)(2)	(X)
Ecografia transvaginale	X(3)	X(3)	(X)(3)	(X)(3)	(X)
Prelievi biomarkers	X(4)		X	X	
Esami ematici	X	X		X	X
Note mediche	(X)	(X)	(X)	(X)	X
Sintomi/ Reazioni avverse	X(5)	X		X	X
X= obbligatorio; (X)= facoltativo, a discrezione dello sperimentatore					

(1) Visite a 6, 18, 30 mesi possono essere effettuate anche da personale infermieristico di ricerca addestrato o da ricercatori clinici coordinati da un medico dello studio

(2) In caso di mammogramma denso, l'ecografia al seno è consigliata

(3) L'ecografia transvaginale è a discrezione del medico e sarà ripetuta in caso del manifestarsi di sanguinamenti imprevisti

(4) Il prelievo ematico per la concentrazione di farmaco e biomarcatori sarà eseguito alla visita basale, 12 mesi, 36 mesi, nel caso di uscita anticipata dallo studio per qualsiasi motivo o a conclusione del trattamento dopo tre anni; campioni di sangue intero per le determinazioni dei polimorfismi genetici (DNA genomico) alla visita basale, alla conclusione del trattamento (per qualsiasi motivo) e a conclusione dello studio dopo i due anni di follow up

(5) Eventuali sintomi preesistenti vanno segnalati in CRF

9.0 EVENTI AVVERSI

9.1 Definizioni

Evento avverso (EA): è qualsiasi evento medico indesiderato in un paziente o soggetto incluso in una sperimentazione clinica e che non ha necessariamente un rapporto causale con il trattamento.

Reazione avversa al farmaco (ADR): Le reazioni tossiche o impreviste dovute al farmaco devono essere considerate *Reazioni avverse al farmaco.*

Tutti gli eventi avversi devono essere annotati sull'apposita sezione della CRF rilevando il grado di tossicità secondo i CTCAE v3.0 e la correlazione al farmaco in studio.

9.2 Follow-up

Tutti gli eventi avversi rilevati saranno aggiornati e seguiti secondo le linee guide di buona pratica clinica.

9.3 Eventi avversi seri (SAE)

Un evento avverso serio è definito come un evento che incontra almeno uno dei seguenti criteri:

- Morte del paziente;
- Causa minaccia immediata per la sopravvivenza stessa del paziente;
- Porta e/o allunga l'ospedalizzazione del paziente;
- Causa disabilità permanente;
- Qualsiasi forma di cancro.

Il cancro alla mammella non sarà considerato come evento avverso serio in quanto è l'obiettivo principale dello studio.

Ogni investigatore garantirà che tutte le informazioni relative ai SAE siano annotate sull'apposita scheda di registrazione e notificate al centro coordinatore entro 24 ore da quando il ricercatore responsabile ne è venuto a conoscenza per la prima volta (tel: 010.5634580/4212; fax: 010.57481402), e che successive informazioni pertinenti siano comunicate entro otto giorni dalla prima segnalazione.

Una volta all'anno, per tutta la durata della sperimentazione clinica, il ricercatore responsabile fornirà ai Comitati Etici un elenco di tutti i SAE e tutte le SUSAR (Suspected Unexpected Serious Adverse Reaction, ossia sospetta e inattesa grave reazione avversa) osservati nel corso dell'intero periodo.

10.0 METODI STATISTICI

10.1 Dimensione del campione

Questo studio clinico randomizzato, di fase III, in doppio cieco, di chemioprevenzione, ha lo scopo di verificare se il trattamento con tamoxifene al dosaggio di 5 mg al giorno per 3 anni risulta efficace, rispetto a nessun trattamento, nel ridurre l'incidenza di cancro mammario invasivo e di carcinoma duttale in situ della mammella, in donne precedentemente operate per neoplasia intraepiteliale lobulare (LIN 2, 3) e duttale (DIN, esclusa DIN 1a) con stato recettoriale ER-positivo o sconosciuto.
In linea con i dati di letteratura (Fisher B et al., Lancet 1999, 353:1993-2000; Fisher B et al., JNCI 1998, 90:1371-1388), nel gruppo di controllo il tasso annuale atteso di incidenza di cancro mammario invasivo e di carcinoma duttale in situ della mammella dovrebbe essere pari al 20/1000. Assumendo una riduzione del 50% (HR=0.50) dell'incidenza di cancro nelle pazienti trattate con tamoxifene, al fine di garantire allo studio una potenza pari all'80%, con un livello di significatività ad una coda pari al 5%, sarà necessario osservare complessivamente 55 eventi (diagnosi di cancro mammario invasivo o di carcinoma duttale in situ). Con un rapporto di assegnazione alla randomizzazione pari a 1:1, un periodo di arruolamento della durata di 5 anni ed un periodo di follow-up minimo di 2 anni, si stima che sia necessario reclutare 1400 pazienti in modo da osservare al termine del periodo di follow-up i 55 eventi necessari. Ciò corrisponde ad un tasso di arruolamento annuale di circa 465 donne, mensile pari a circa 40 donne.
Il calcolo della dimensione campionaria è stato fatto con l'assunzione di un arruolamento uniforme, in assenza di effetto "time-lag" del tamoxifene e ipotizzando una persistenza del suo effetto dopo la sospensione della sua somministrazione.
Considerando un reclutamento inferiore, per aumentare la potenza statistica e poter quindi ottenere una valutazione clinica significativa, lo studio verrà analizzato insieme allo studio IEO007, già concluso e che ha utilizzato la stessa dose di tamoxifen in donne con DIN o LIN in premenopausa.

10.2 Popolazioni in studio

Per le analisi descrittive e statistiche pianificate si utilizzeranno, a seconda dei casi in seguito specificati, le seguenti popolazioni:
Intention-To-Treat (ITT): questa popolazione include tutte le pazienti randomizzate. Tutti i soggetti verranno inclusi nel gruppo di trattamento al quale erano stati assegnati al momento della randomizzazione, indipendentemente dalla compliance e dal tipo di trattamento effettivamente ricevuto. Tutte le donne saranno dovranno essere seguite fino al termine dello studio anche nel caso di interruzione del trattamento.

Safety: questa popolazione include tutti le pazienti randomizzate che hanno ricevuto almeno una dose di farmaco. Il gruppo di trattamento di appartenenza verrà definito sulla base del trattamento effettivamente ricevuto.

10.3 Piano di analisi statistica

Confronto delle caratteristiche alla randomizzazione

La distribuzione delle pazienti sulla base delle loro caratteristiche cliniche e biologiche alla randomizzazione verrà riportata in maniera descrittiva per i due gruppi di trattamento. Il test esatto di Fisher verrà utilizzato per confrontare la distribuzione delle pazienti non eleggibili nei due gruppi di trattamento.

Analisi di efficacia

<u>Endpoint primario</u>

L'incidenza di cancro mammario invasivo e di carcinoma duttale in situ della mammella, in donne precedentemente operate per neoplasia intraepiteliale lobulare (LIN 2, 3) e duttale (DIN, esclusa DIN 1a) con stato recettoriale ER-positivo o sconosciuto, rappresenta la variabile di efficacia principale dello studio.

L'analisi principale di efficacia sarà basata sulla popolazione ITT. La stima di effetto verrà espressa in termini di stima puntuale del rapporto fra gli hazard (hazard nel gruppo di controllo / hazard nel gruppo sperimentale) e del relativo intervallo di confidenza al 95% (ad una coda). La stima verrà calcolata mediante il modello semi-parametrico ad hazard proporzionali di Cox ed il test di significatività utilizzato sarà il likelihood ratio test. A fini descrittivi verranno stimate le curve di incidenza cumulativa di cancro mammario invasivo e di carcinoma duttale in situ della mammella utilizzando la tecnica di Kaplan-Meier, e verranno riportati i valori della probabilità cumulativa di sviluppare cancro a 3 anni dalla randomizzazione, con il relativo intervallo di confidenza al 95% (ad una coda).

<u>Analisi per sottogruppo</u>

Sulla variabile di efficacia principale dello studio le principali analisi per sottogruppo che verranno effettuate saranno: casi incidenti e casi prevalenti, pazienti con DIN e con LIN, stato recettoriale ER+ e ER sconosciuto. Preventivamente alle analisi per sottogruppo si utilizzerà il modello semi-parametrico ad hazard proporzionali di Cox per studiare l'effetto di interazione fra il fattore trattamento e ciascun fattore di stratificazione. Solo nel caso in cui il termine di interazione mostri una significatività statistica $\geq$10% (test a due code) si potrà procedere alla stima dell'indicatore di effetto in ciascuno strato identificato dal fattore di stratificazione.

Tutte le analisi per sottogruppo si baseranno sulla popolazione ITT.

Analisi per la safety

L'analisi per la safety verrà effettuata sulla popolazione per la safety.

Tutti gli eventi avversi seri verranno periodicamente riportati e discussi con l'investigatore principale e tutti gli sperimentatori coinvolti. Come linea guida generale, si dovrebbe prendere in considerazione l'eventuale stop precoce del trial solo se si verificasse una differenza tra i bracci pari a tre volte la deviazione standard per alcuni importanti eventi (quali il cancro endometriale e/o eventi tromboembolici).

Analisi intermedie

Non viene pianificata alcuna analisi intermedia di efficacia.

11.0 BIBLIOGRAFIA

1 Allred DC, Anderson SJ, Paik S, Wickerham DL, et al: Adjuvant Tamoxifen Reduces Subsequent Breast Cancer in Women With Estrogen Receptor-Positive Ductal Carcinoma in Situ: A Study Based on NSABP Protocol B-24. *J Clin Oncol*. 2012 Apr 2.

2 Wapnir IL, Dignam JJ, Fisher B, Mamounas EP et al: Long-term outcomes of invasive ipsilateral breast tumor recurrences after lumpectomy in NSABP B-17 and B-24 randomized clinical trials for DCIS. *J Natl Cancer Inst*. 2011 Mar 16;103(6):478-88.

3 Cuzick J, Sestak I, Pinder SE, et al: Effect of tamoxifen and radiotherapy in women with locally excised ductal carcinoma in situ: long-term results from the UK/ANZ DCIS trial.
Lancet Oncol. 2011 Jan;12(1):21-9. Epub 2010 Dec 7.

4 Virnig BA, Tuttle TM, Shamliyan T, Kane RL
Ductal carcinoma in situ of the breast: a systematic review of incidence, treatment, and outcomes.*J Natl Cancer Inst*. 2010 Feb 3;102(3):170-8.

5 Kelloff GJ, Lippman SM, Dannenberg AJ et al: Progress in chemoprevention drug development: the promise of molecular biomarkers for prevention of intraepithelial neoplasia and cancer--a plan to move forward. *Clin Cancer Res* 2006: 12: 3661-97.

6 Fisher B, Costantino JP, Wickerham DL et al. Tamoxifen for prevention of breast cancer: report of the National Surgical Adjuvant Breast and Bowel Project P-1 Study. *J Natl Cancer Inst* 1998; 90: 1371-88.

7 Fisher B, Dignam J, Wolmark N et al. Tamoxifen in treatment of intraductal breast cancer: National Surgical Adjuvant Breast and Bowel Project B-24 randomised controlled trial. *Lancet* 1999; 353: 1993-2000.

8 Houghton J, George WD, Cuzick J, et al. Radiotherapy and tamoxifen in women with completely excised ductal carcinoma in situ of the breast in the UK, Australia, and New Zealand: randomised controlled trial. *Lancet* 2003; 362: 95-102.

9 Early Breast Cancer Trialists' Collaborative Group. Tamoxifen for early breast cancer: an overview of the randomised trials. *Lancet* 1998; 351: 1451-67.

10 Fallowfield L, Fleissig A, Edwards R, et al. Tamoxifen for the prevention of breast cancer: psychosocial impact on women participating in two randomized controlled trials. *J Clin Oncol* 2001; 19: 1885-92.

11 Cuzick J, Forbes JF, Sestak I, et al. International Breast Cancer Intervention Study I Investigators. Long-term results of tamoxifen prophylaxis for breast cancer--96-month follow-up of the randomized IBIS-I trial. *J Natl Cancer Inst* 2007; 99: 272-82.

12 Rutqvist LE, Johansson H, Signomklao T, Johansson U, Fornander T, Wilking N. Adjuvant tamoxifen therapy for early stage breast cancer and second primary malignancies. Stockholm Breast Cancer Study Group. *J Natl Cancer Inst* 1995; 87: 645-651.

13 Jordan VC. Tamoxifen: too much for a good thing? *J Clin Oncol* 1999; 17: 2629-30.

14 Tavassoli FA. Ductal intraepithelial neoplasia of the breast. Virchows Arch 2001; 438: 221-7.

15 Tavassoli FA. Ductal carcinoma in situ: introduction of the concept of ductal intraepithelial neoplasia. *Mod Pathol* 1998;11:140-54.

16 Bratthauer GL, Tavassoli FA. Lobular intraepithelial neoplasia: previously unexplored aspects assessed in 775 cases and their clinical implications. Virchows Arch. 2002 Feb;440(2):134-8.

17 Tavassoli FA. Lobular and ductal intraepithelial neoplasia. Pathologe. 2008 Nov;29 Suppl 2:107-11.

18 Frykberg ER. Mammary Intraepithelial Neoplasia: A Translational Classification System for the Intraductal Epithelial Proliferations. The Breast Journal, Volume 3, Number 1, 1997 48-58.

19 Guerrieri-Gonzaga A, Botteri E, Rotmensz N, et al. Ductal Intraepithelial neoplasia: postsurgical outcome for 1,267 women cared for in one single institution over 10 years. Oncologist. 2009 Mar;14(3):201-12.

20 Center for Drug Evaluation and Research (03/08/2005). "Tamoxifen Information: reducing the incidence of breast cancer in women at high risk". U.S. Food and Drug Administration. http://www.fda.gov/cder/news/tamoxifen/.

21 NCCN Practice Guidelines in Oncology, Breast Cancer v. 1.2009. http://www.nccn.org/professionals/physician_gls/f_guidelines.asp.

22 Italian Association of Medical Oncology (AIOM) guidelines for breast neoplasms: http://www.aiom.it/C_Common/Download.asp?file=/doc/upload/Attivita_Scientifica/Linee_Guida/LG_Mammella_AIOM08.pdf.

23 Maltoni C, Minardi F, Belpoggi F, Pinto C, Lenzi A, Filippini F. Experimental results on the chemopreventive and side effects of tamoxifen using a human-equivalen animal model. In: Maltoni C, Soffritti M, and Davis W editors. The scientific bases of cancer chemoprevention. Amsterdam: Elservier Science 1996, 197-217.

24 Bergman L, Beelen ML, Gallee MP, et al. Risk and prognosis of endometrial cancer after tamoxifen for breast cancer. Comprehensive Cancer Centres' ALERT Group. Assessment of Liver and Endometrial cancer Risk following Tamoxifen. *Lancet* 2000, 356, 881-887.

25 Breuer B, Wallenstein S, Anderson R. Effect of tamoxifen on bone fractures in older nursing home residents. *J Am Geriatr Soc* 1998, 46, 968-972.

26 Decensi A, Maisonneuve P, Rotmensz N, et al. Effect of tamoxifen on venous thromboembolic events in a breast cancer prevention trial. Circulation 2005; 111: 650–6.

27 Sivridis E, Giatromanolaki A: Proliferative activity in postmenopausal endometrium: The lurking potential for giving rise to an endometrial adenocarcinoma. J Clin Pathol 2004; 57: 840-844.

28 Day R, Ganz PA, Costantino JP, et al: Health related quality of life and tamoxifen in breast cancer prevention: A report from the National Surgical Adjuvant Breast and Bowel Project P-1 Study. J Clin Oncol 17:2659-2669, 1999.

29 Kamphuisen PW, Eikenboom JC, Vos HL, et al: Increased levels of factor VIII and fibrinogen in patients with venous thrombosis are not caused by acute phase reactions. Thromb Haemost 81:680-683, 1999.

30 Guerrieri-Gonzaga A, Robertson C, Bonanni B, et al. A Preliminary results on safety and activity of a randomized, double-blind, 2 x 2 trial of low-dose tamoxifen and fenretinide for breast cancer prevention in premenopausal women. *J Clin Oncol* 2006; 24: 129-35.

31 Decensi A, Bonanni B, Guerrieri-Gonzaga A et al. Biologic activity of tamoxifen at low doses in healthy women. *J Natl Cancer Inst* 1998, 90, 1461-1467.

32 Decensi A, Gandini S, Guerrieri-Gonzaga A et al. Effect of blood tamoxifen concentrations on surrogate biomarkers in a trial of dose reduction. *J Clin Oncol* 1999, 17, 2633-2638.

33 Decensi A, Robertson C, Viale G et al. A randomized trial of low-dose tamoxifen on breast cancer proliferation and blood estrogenic biomarkers. J Natl Cancer Inst 2003, 95, 779-90.

34 Decensi A, Veronesi U, Miceli R, et al: Relationships between plasma insulin-like growth factor-I and insulin-like growth factor binding protein-3 and second breast cancer risk in a prevention trial of fenretinide. *Clin Cancer Res 2003;* 9: 4722-9.

35 Hankinson SE, Willett WC, Colditz GA, et al: Circulating concentration of insulin-like growth factor-I and risk of breast cancer. Lancet 1998; 351:1393-6.

36 Johansson H, Baglietto L, Guerrieri-Gonzaga A, et al: Factors associated with circulating levels of insulin-like growth factor-I and insulin-like growth factor binding protein-3 in 740 women at risk for breast cancer. Breast Cancer Res Treat 88:63-73, 2004.

37 Toniolo P, Bruning PF, Akhmedkhanov A, et al: Serum insulin-like growth factor-I and breast cancer. Int J Cancer 88:828-32, 2000.

38 Torrisi R, Pensa F, Orengo MA, et al: The synthetic retinoid fenretinide lowers plasma insulin-like growth factor I levels in breast cancer patients. Cancer Res 53:4769-71, 1993

39 Lien EA, Solheim E, Lea OA, et al: Distribution of 4-hydroxy-N-desmethyltamoxifen and other tamoxifen metabolites in human biological fluids during tamoxifen treatment Cancer Res 49:2175-83, 1989.

40 Stanton AL, Bernaards CA, Ganz PA. The BCPT Symptom Scales: a measure of physical symptoms for women diagnosed with or at risk for breast cancer. *Journal of the National Cancer Institute* 2005; 97: 448-456.

41 Sloan A, Loprinzi CL, Novotny PL, et al. Methodologic Lessons Learned From Hot Flash Studies. *J Clin Oncol* 2001, 19, 4280-4290.

42 Goetz MP, Rae JM, Suman VJ, et al: Pharmacogenetics of tamoxifen biotransformation is associated with clinical outcomes of efficacy and hot flashes. J Clin Oncol 23:9312-8, 2005.

43 Bonanni B, Macis D, Maisonneuve P, et al: Polymorphism in the CYP2D6 tamoxifen metabolizing gene influences clinical effect but not hot flashes: data from the Italian Tamoxifen Trial. *J Clin Oncol* 24:3708-9; author reply 3709, 2006.

44 Goetz MP, Knox SK, Suman VJ, et al. The impact of cytochrome P450 2D6 metabolism in women receiving adjuvant tamoxifen Breast Cancer Res Treat. 2007 Jan;101(1):113-21.

45 Serrano D. Abstract - No. 503 2007 ASCO Annual Meeting - Category: Breast Cancer--Local-Regional and Adjuvant Therapy - Adjuvant Therapy.

STUDIO RANDOMIZZATO PLACEBO CONTROLLATO DI FASE III CON TAMOXIFEN A BASSE DOSI IN DONNE CON NEOPLASIA INTRAEPITELIALE DELLA MAMMELLA.

STUDIO TAM-01

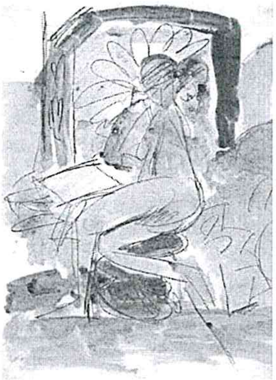

Vasomotor symptoms questionnaire

Stanton AL, Bernaards CA, Ganz PA. The BCPT Symptom Scales: a measure of physical symptoms for women diagnosed with or at risk for breast cancer. Journal of the National Cancer Institute 2005; 97:448-456.

Questionario per la Paziente

Breve Questionario sulla Valutazione dei Sintomi Vasomotori

PAZIENTE |__|__|.|__|__|

(numero confezione)

Visita ___________

DATA |__|__|.|__|__|.|__|__|__|__|

DISTURBI QUOTIDIANI RISCONTRATI NELLE ULTIME 4 SETTIMANE

Siamo interessati nel conoscere alcuni sintomi/disturbi quotidiani che ha riscontrato ultimamente: per ciascuno dei seguenti disturbi indichi quanto è stato fastidioso **nelle ultime 4 settimane** (*cerchiando un solo numero corrispondente per ogni disturbo; se non ha avuto il disturbo, la preghiamo di cerchiare lo zero, "per niente"*).

Durante le **ultime 4 settimane** a che livello è stata disturbata da:	Per Niente	Legger-mente	Moderata -mente	Abbasta-nza	Estrema-mente
1. Vampate di calore..	0	1	2	3	4
2. Nausea..	0	1	2	3	4
3. Vomito..	0	1	2	3	4
4. Difficoltà nel mantenere lo stimolo all'urina durante il riso o il pianto..	0	1	2	3	4
5. Difficoltà nel mantenere lo stimolo all'urina in altre situazioni..	0	1	2	3	4
6. Secchezza vaginale..	0	1	2	3	4
7. Dolore durante il rapporto sessuale........................	0	1	2	3	4
8. Dolori e/o indolenzimento generale.......................	0	1	2	3	4
9. Dolori alle articolazioni......................................	0	1	2	3	4
10. Rigidità muscolare..	0	1	2	3	4
11. Aumento di peso...	0	1	2	3	4
12. Senso di insoddisfazione per il proprio aspetto fisico...	0	1	2	3	4
13. Perdita di memoria	0	1	2	3	4
14. Sudorazioni notturne......................................	0	1	2	3	4
15. Difficoltà di concentrazione...............................	0	1	2	3	4
16. Facilità di distrazione.....................................	0	1	2	3	4
17. Gonfiore alle braccia (linfedema)........................	0	1	2	3	4
18. Ridotta mobilità del braccio vicino al seno operato.....	0	1	2	3	4

Hot flashes evaluation

Sloan A, Loprinzi CL, Novotny PL, et al. Methodologic Lessons Learned From Hot Flash Studies. J Clin Oncol 2001;19:4280-4290.

Studio TAM-01 questionario –V.1.4 del 12/01/09

Valutazione dell'intensità delle VAMPATE DI CALORE:

Per descrivere il grado di intensità delle vampate di calore, La preghiamo di riferirsi ai seguenti esempi di sintomi che sono stati forniti da donne che, come Lei, hanno partecipato a studi clinici e a cui è stato chiesto di descriverne le caratteristiche. Tali esempi la possono aiutare nel definire meglio il suo sintomo e nel farlo rientrare in una delle seguenti **4 categorie di intensità**: **lieve, moderata, severa, molto severa.**

LIEVE
-*Durata*: meno di 5 minuti;
-*Sintomi fisici*: calore, sensazione di disagio, rossore in viso;
-*Stati d'animo*: lieve sensazione di ansia ed inadeguatezza nell'affrontare un evento imprevisto;
-*Azioni*: di solito nulla di particolare.

MODERATA
-*Durata*: fino a 15 minuti.
-*Sintomi fisici*: calore alla testa, al collo, alle orecchie o a tutto il corpo; tensione muscolare; pelle umida e sudore diffuso; cambiamenti nel ritmo dei battiti cardiaci; bocca secca.
-*Stati d'animo*: irritabilità, agitazione e stanchezza, sensazione di perdita di energie e imbarazzo di fronte agli altri.
-*Azioni*: qualche volta capita di svegliarsi d'improvviso durante la notte, di avere bisogno d'aria, di scoprirsi, di svestirsi o di utilizzare vestiti più leggeri, di bere acqua, di aprire le finestre anche quando fuori c'è molto freddo.

SEVERA
-*Durata*: fino a 20 minuti;
-*Sintomi fisici*: sensazione di calore intenso, come di una fornace o di un fuoco; cambiamenti nel ritmo cardiaco e/o aumento del battito; sensazione di svenimento; mal di testa; sudore intenso; debolezza, formicolio, sensazione di pizzichi sulla pelle; pesantezza al torace.
-*Stati d'animo*: imbarazzo, ansia, paura di avere un attacco di panico.
-*Azioni*: interruzione dell'attività intrapresa fino a quel momento, sveglie notturne con bisogno di levare le coperte; necessità di svestirsi, di aprire le finestre, di abbassare la temperatura di casa; utilizzo frequente di ventilatori.

MOLTO SEVERA:
-*Durata*: fino a 45 minuti;
-*Sintomi fisici*: sensazione calore intenso come di ebollizione; sudorazione molto intensa, difficoltà a respirare, formicolio, stordimento, tensione muscolare e/o crampi alle gambe, cambiamenti nel ritmo cardiaco e/o aumento del battito, sensazione di malessere allo stomaco.
-*Stati d'animo*: stress intenso, bisogno di scappare via, sensazione di non riuscire a far nulla.
-*Azioni*: frequenti sveglie notturne, bisogno di cambiare lenzuola e pigiama, voglia di fare una doccia fredda, di tenere del ghiaccio sulla pelle.

La preghiamo di compilare la seguente tabella circa il **numero** e **l'intensità** delle vampate di calore che ha riscontrato **durante l'ultima settimana**:

	1° giorno*	2° giorno	3° giorno	4° giorno	5° giorno	6° giorno	7° giorno
Numero di vampate al giorno, per grado di intensità: ***lieve, moderata, severa, molto severa***	___lieve<br>___moderata<br>___severa<br>___molto sev.	___lieve<br>___moderata<br>___severa<br>___molto sev.	___lieve<br>___moderata<br>___severa<br>___molto sev.	___lieve<br>___moderata<br>___severa<br>___molto sev.	___lieve<br>___moderata<br>___severa<br>___molto sev.	___lieve<br>___moderata<br>___severa<br>___molto sev.	___lieve<br>___moderata<br>___severa<br>___molto sev.
Numero TOTALE di vampate al giorno							

* Un giorno deve essere considerato di 24 ore, comprensivo quindi di notte e giorno.

STUDIO TAM-01

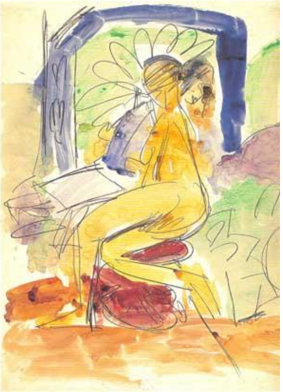

PRELIEVI

Blood withdrawal protocol

Modulistica e raccolta dati

- I PRELIEVI VANNO EFFETTUATI ALLE VISITE:

  basale, 12 mesi(VISITA3), 36 mesi(VISITA 7) o all'Uscita dallo studio per qualsiasi motivo, e FU 2° anno

- le etichette contenute nelle buste delle provette e corrispondenti ai vari prelievi (basale, 12 mesi, 36 mesi, uscita dallo studio e FU 2° anno) vanno applicate nell'apposita sezione del FORM P1
- va applicata l'etichetta anche per la **farmacogenetica** FORM P1

- per richiedere il rifornimento delle provette compilare il FORM P2 e inviarlo secondo istruzioni
- per l'esecuzione e lo stoccaggio dei prelievi seguire le istruzioni allegate

FORM P3

CODICE ALFABETICO* |__|__|__| CONFEZIONE N.|__|__|.|__|__|

*Riportare il codice alfabetico scelto in fase di randomizzazione

P1. PRELIEVI EMATICI BIOMARKERS E FARMACOGENETICA

VISITA 1 (BASALE)

Prelievo per **Biomarkers**

Data del prelievo (gg/mm/aaaa)|__|__|/|__|__|/|__|__|__|__|

Se non effettuato, motivo:

APPLICARE IL CODICE A BARRE CORRISPONDENTE AL PRELIEVO

VISITA 3 (12 MESI)

Prelievo per Biomarkers

Data del prelievo (gg/mm/aaaa)|__|__|/|__|__|/|__|__|__|__|

Se non effettuato, motivo:

APPLICARE IL CODICE A BARRE CORRISPONDENTE AL PRELIEVO

VISITA 7 (36 MESI)

Prelievo per Biomarkers

Data del prelievo (gg/mm/aaaa)|__|__|/|__|__|/|__|__|__|__|

Se non effettuato, motivo:

APPLICARE IL CODICE A BARRE CORRISPONDENTE AL PRELIEVO

FINE TRATTAMENTO SPERIMENTALE

(uscita dallo studio per qualsiasi motivo)

Prelievo per **Biomarkers** (obbligatorio all'uscita dallo studio)

❑No ❑ Si, Data del prelievo (gg/mm/aaaa)|__|__|/|__|__|/|__|__|__|__|
Se non effettuato, motivo:

APPLICARE IL CODICE A BARRE CORRISPONDENTE AL PRELIEVO

PRELIEVO FARMACOGENETICA

❑No ❑ Si, Data del prelievo (gg/mm/aaaa)|__|__|/|__|__|/|__|__|__|__|

APPLICARE IL CODICE A BARRE CORRISPONDENTE AL PRELIEVO

Se non effettuato, motivo:

FOLLOW UP 2° ANNO

Prelievo di Sangue EDTA dopo 2 anni dal termine del trattamento

❑No ❑ Si, Data del prelievo (gg/mm/aaaa)|__|__|/|__|__|/|__|__|__|__|

Se non effettuato, motivo:

APPLICARE IL CODICE A BARRE CORRISPONDENTE AL PRELIEVO

Mod_Richiesta k lab_V.3 del 16/10/2014

P2 - MODULO RICHIESTA FORNITURA KIT

STUDIO	TAM-01	Denominazione Centro
Numero Centro		
Sperimentatore Responsabile		

Completare il form in ogni sua parte ed inviarlo per fax o per mail a Harriet Johansson / Debora Macis

n° FAX: 02.-9437 9225

divisione.prevenzionegeneticaoncologica@ieo.it

ATTENZIONE!!!

Inviare la richiesta con almeno 2 settimane di anticipo cercando di prevedere con anticipo il consumo futuro di un periodo di circa 6 mesi

n° Kits Richiesti	Prelievo Basale/36mesi	Prelievo a 12 mesi	Prelievo a FU 2° anno
	n° |__|__|__|	n° |__|__|__|	n° |__|__|__|
Nome del richiedente			
Contatto (telefono/mail)			
Data e Firma			

	**INDIRIZZO DI SPEDIZIONE DEI KITS**	
Istituzione		
Via/P.zza		n°

CAP		Città	
Telefono		Fax	
Note			

IEO - Divisione di Prevenzione Genetica e Oncologica – Via Ripamonti 435, 20141 - Milano
Harriet Johansson e Debora Macis - Tel 02 94372654 - 02 57489379

P3 – Guida rapida per raccolta e processamento sangue e siero

La paziente deve essere a digiuno e il prelievo possibilmente eseguito tra le 8 e le 10 del mattino.

Il prelievo di siero per biomarcatori circolanti prevede la raccolta di circa 15 ml di sangue e va effettuato alla visita basale, 12 mesi e 36 mesi (o all'uscita dallo studio); Per esempio: utilizzare 3 provette vacutainer da 4.9 ml con biglie contenenti un attivatore della coagulazione (S-monovette della Sarstedt)].

Processamento Siero:

- Lasciare nelle provette da prelievo a temp. amb. per circa 30 min.;
- Centrifugare a 3000 rpm (circa 1350 x g) per 10 min. Al termine della centrifugazione troverete uno strato superiore di colore paglierino (siero), e uno strato inferiore rosso scuro (pellet di cellule ematiche);
- Aspirare il siero senza sfiorare lo strato di cellule ematiche sottostanti e aliquotarlo (circa 1,5 ml/microprovetta) in **5 microprovette** e chiudere con **tappo trasparente**, stringendolo forte.

Il prelievo di sangue EDTA-trattato prevede la raccolta di circa 5 ml di sangue da eseguirsi sia al **basale**, **36 mesi** e alla fine dei **2 anni di FU** che seguono il termine del trattamento;

Processamento sangue EDTA trattato:

- Le 2 provette vanno agitate delicatamente con la mano, o mettendole sulla ruota dell'emocromo per circa 5 minuti in maniera da miscelare bene il sangue;
- Una volta miscelato, aliquotare (circa 1,3 ml/microprovetta) in **4 microprovette** e chiudere con **tappo rosso**, stringendolo forte;

Etichettatura e storaggio:

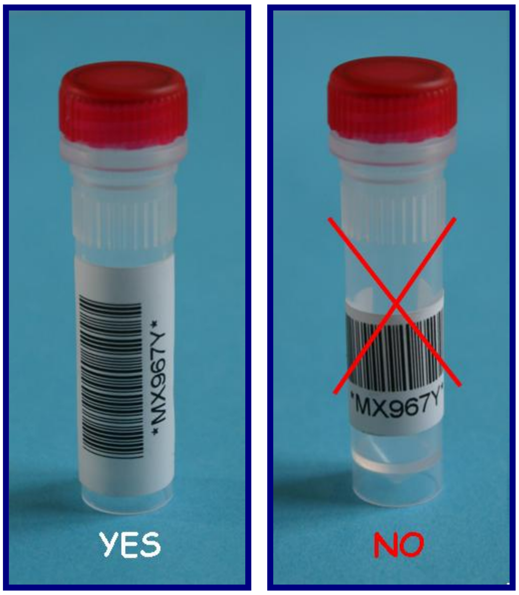

1. Attaccare le etichette piccole sulle microprovette in senso verticale, come illustrato nella foto a fianco. E' consigliabile etichettare sia la provette di raccolta sangue che le microprovette prima di procedere al prelievo e tenerli assieme in modo da non confondere prelievi provenienti da pazienti diversi.
2. Completare l'etichetta grande con i dati relativi alla paziente: ID paziente (corrisponde al No confezione in basso al centro sulla scheda di randomizzazione), iniziali della paziente (in ordine: primo nome, eventuale secondo nome, cognome; se doppio indicare le 2 iniziali), data di nascita (gg/mm/aaaa), data prelievo (gg/mm/aaaa), tempo: cerchiare il tempo corrispondente.
3. Tutte le microprovette vanno riposte nel cryobox fornito insieme ai kit e conservate in freezer a -80°C (se non possibile, a -20°C). Riporre la patient card nel sacchettino piccolo fornito con il kit e compilare la scheda

collocazione campioni.

4. Si consiglia di tenere traccia della collocazione dei campioni nel congelatore, e di monitorare il corretto funzionamento del freezer.

Ordinare nuovi kit con almeno 2 settimane di preavviso a:

Istituto Europeo di Oncologia
Divisione di Prevenzione e Genetica Oncologica
Att.ne Harriet Johansson (Tel. 02-94372654)/ Debora Macis (Tel. 02-57489379)
Via Ripamonti 435, 20141 Milano
Fax: +39 02 94379225
e-mail: divisione.prevenzionegeneticaoncologica@ieo.it

Supplementary Material 2

Association of CYP2D6 genotype and tamoxifen metabolites with breast cancer recurrence in a low-dose trial

by A. DeCensi et al

Contents

STEERING COMMITTEE 4

INDEPENDENT DATA MONITORING COMMITTEE 4

SUPPLEMENTARY METHODS 4

STUDY SITES 4

Coordinator Center 4

Recruiting Sites and Collaborators 4

TRIAL OBJECTIVES 6

Primary objective 6

Secondary objectives 6

PATIENT SELECTION CRITERIA 7

Inclusion Criteria 7

Exclusion Criteria 7

STUDY TREATMENT 8

Dose Regimen 8

Experimental treatment 8

Method of Randomization 9

Experimental treatment administration 9

Experimental treatment discontinuation 9

Concomitant drugs 11

PHARMACEUTICAL INFORMATION 11

Study Agents 11

Agent Distribution 11

Formulation 12

Packaging and Labeling 12

TRIAL ASSESSMENTS 12

Instrumental Assessments 12

Centralized Laboratory Exams 13

TRIAL PROCEDURES 14

Baseline visit 14

Evaluations at 6, 12, 18, 24, 30, 36 month visits 14

Follow-up plan 15

Mammography 15

Off-agent criteria 16

Off-study criteria 16

Study Termination 17

STUDY PROCEDURES FLOW-CHART 18

STATISTICAL ANALYSIS PLAN 19

Sample size 19

Study population 19

Comparison of characteristics at the time of randomization ("baseline") 20

Analysis of efficacy 20

Subgroup analyses 20

REFERENCES 21

STEERING COMMITTEE

Andrea DeCensi, MD, E.O. Ospedali Galliera, Genoa, Italy, Wolfson Institute of Preventive Medicine, Queen Mary University of London, London, UK; **Bernardo Bonanni**, MD, IRCCS European Institute of Oncology, Milan, Italy; **Luca Boni**, MD, AOU Careggi, Firenze, Italy; **Matteo Puntoni**, PhD, E.O. Ospedali Galliera, Genoa, Italy.

INDIPENDENT DATA MONITORING COMMITTEE

Chair: **Alberto Costa**, MD, Breast Unit, IOSI, Lugano, Switzerland; European School of Oncology (ESO), Milan, Italy; Members: **Sara Gandini**, PhD, Molecular and Pharmacoepidemiology Unit, European Institute of Oncology IRCCS, Milan, Italy, **Paolo Pronzato**, MD, Department of Medical Oncology, IRCCS Azienda Ospedaliera Universitaria San Martino - Istituto Nazionale per la Ricerca sul Cancro, Genoa, Italy.

SUPPLEMENTARY METHODS

STUDY SITES

Coordinator Center

E.O. Galliera, Division of Medical Oncology, Principal Investigator: **Andrea DeCensi;** Collaborators: **D'Amico M, Caviglia S, Briata IM, Buttiron Webber T.**

Patients were enrolled from 18 sites across Italy. 4 sites were prematurely closed due to low enrollment rate.

Recruiting Sites and Collaborators

IRCCS European Institute of Oncology, Milan: **B. Bonanni, M. Lazzeroni, D. Serrano, A. Guerrieri Gonzaga, M Cazzaniga**;

IRCCS Istituto nazionale per lo studio e la cura dei tumori, "Fondazione Pascale" Naples: G. **D’Aiuto, F. Avino**;

A. O. Universitaria Policlinico di Modena, Modena: **L. Cortesi**;

A.O. Universitaria S. Giovanni Battista - “Le Molinette”, Turin: **A. Ponti, M. Donadio**;

Ospedale SS Antonio e Margherita-ASL AL, Tortona: **M.G. Pacquola**;

IRCCS Istituto Scientifico Romagnolo per lo Studio e la Cura dei Tumori (I.R.S.T.), Meldola: **F. Falcini**;

ULSS 6 Vicenza, Vicenza: **M. Gulisano**;

IRCCS Istituto tumori “G. Paolo II”, Bari: **C. D’Amico, M. Digennaro**;

Ospedale Santa Maria delle Croci, Ravenna: **G. Cruciani, A. Tienghi**;

Ospedale Bernardino Ramazzini, Carpi: **K. Cagossi**;

Asst Sette Laghi, Varese: **G Giardina, G. Pinotti**;

Fondazione per la ricerca e la Cura dei Tumori “T. Campanella”; Catanzaro: **G. Di Paola, Nuccia Renne**;

IRCCS Fondazione Salvatore Maugeri, Pavia: **L. Regolo, S. Fissi, A. De Simone**;

IRCCS Azienda Ospedaliera Universitaria San Martino - Istituto Nazionale per la Ricerca sul Cancro, Genoa: **G. Canavese, G. Iacono**;

IRCCS Azienda Ospedaliera Universitaria San Martino - Istituto Nazionale per la Ricerca sul Cancro, Genoa: **D. Friedman**.

TRIAL OBJECTIVES

Primary objective

Primary objective of the study is to assess whether tamoxifen at a dose of 5mg per day administered for 3 years reduces the incidence of invasive breast cancer and in situ breast ductal carcinoma (DIN 2 and DIN 3).

Secondary objectives

- Incidence of other non-invasive events (i.e., LCIS, atypical ductal hyperplasia)

- Assessment of tolerability of tamoxifen: incidence of endometrial carcinoma, ovarian cancer, thromboembolic events

- Incidence of fractures, cardiovascular and cardiac arterial events, clinically evident cataract and melanoma

- Mammographic density evaluation

- In a subgroup of patients centralized measurements of circulating levels of tamoxifen and metabolites, IGF-I, IGFBP3, SHBG, CRP and on some SNPs of cytochrome P450 genes involved in the metabolism of tamoxifen CYP2D6 (* 4, * 2, * 5, 6 * and * 7) and CYP2C19.

- Centralized tissue analysis: evaluation of hormone receptors (estrogen and progesterone) and Her-2, of the proliferative index Ki-67 antigen. The cases considered 2+ for Her-2 (moderate complete membrane immunoreactivity) will be submitted to FISH for confirmation.

- Tumor tissue microarray and mutation profile by next generation sequencing were planned pending additional funding.

PATIENT SELECTION CRITERIA

Inclusion Criteria

- Women of age $\geq 18 < 75$ years
- Women operated for lobular (LIN 2 and 3) or ER positive or unknown ductal (DIN 1-3, excluded DIN 1a) intraepithelial neoplasia. Both incident (diagnosis within 12 months) and prevalent cases (diagnosis between previous 12 and 60 months) will be included, upon stratification.
- ECOG performance Status ≤ 1
- Written informed consent

Exclusion Criteria

- Any type of malignancy, with the exclusion of non-melanoma skin cancer;
- Active proliferative disorders of the endometrium such as atypical hyperplasia, history of active endometriosis, unresected polyps, symptomatic myoma;
- Alterations of metabolic, liver, renal and cardiac grade 2 function (NCI criteria grade 2 or higher);
- Any type of retinal disorders or severe cataract or glaucoma;
- Presence of significant risk factors for venous events, including immobilization within the last 3 months for longer than 2 weeks following surgery or trauma, history of phlebitis concomitant to estrogen therapy , deep venous thrombophlebitis or other significant venous thrombotic event (VTE) (pulmonary embolism, stroke, etc.);
- Previous use of tamoxifen, raloxifene or other selective estrogen receptor modulator (SERMs);

- Use of anastrozole or other Aromatase Inhibitors (AI) in the last 12 months for a period longer than or equal to 6 months;
- Anticoagulant therapy in progress (heparin or dicoumarol);
- CYP2D6 inhibitors such as selective serotonin reuptake inhibitors, cimetidine, haloperidol;
- Active infections;
- Severe psychiatric disorders or inability to comply to the protocol procedures.
- Geographical inaccessibility to healthcare or logistic difficulties that may interfere with protocol procedure;
- Pregnant or breastfeeding women;
- Any other factors that, at the investigator's discretion, may contraindicate the use of tamoxifen

STUDY TREATMENT

Dose Regimen

The investigational study drug used in the course of this trial was tamoxifen oral tablets 5 mg/die. In order to avoid any detection bias, the study was planned as double blind.

Patients satisfying eligibility criteria were randomized, with a 1:1 ratio, to receive either tamoxifen (Experimental arm) or placebo (Control arm).

Experimental treatment

Patient and investigators were blinded to study treatment. Tamoxifen/placebo was provided by Doppel Farmaceutici S.r.l, manufactured and labelled as study drug.

Tamoxifen/placebo was self-administered as a single oral tablet of 5 mg/day, up to 36 months, or until disease progression, unacceptable toxicity or physician/patient decision to withdraw, whichever comes first.

Method of Randomization

The assignment of patients to treatment arms was performed using a centralized randomization system, using the minimization algorithm. Each site was provided with a specific identification code and investigators were be able to randomize patients to treatments using individual login name (username) and password that granted access to a study-dedicated randomization web page.Random sequences were generated by Luca Boni according to the Moses-Oakford algorithm. Only the system administrator had access to the allocations sequences. Stratification criteria were center, timing of diagnosis (within 12 vs 12-60 months) and histotype (ADH+DCIS vs. LCIS).

Experimental treatment administration

The Principal Investigators of each local site was responsible for drug administration.

Principal Investigators could delegate other study investigators (MDs, pharmacists, and research nurses) to dispense the agent on their behalf.

At each visit, patients received a 7-month supply (210 tablets) kit of drug/placebo. The Investigator or designee identified the study drug package to dispense to the patient by the medication number assigned to each patient at randomization. Every time that the 7-months supply kit was dispensed to the patient and/or legally acceptable representative, the investigator provided detailed instructions on how to take the study treatment properly, according to the following schedule:

- tamoxifen/placebo should be taken once daily.

Experimental treatment discontinuation

Patients were asked to maintain the full dose throughout the treatment period.

Adverse event were evaluated using the NCI Common Terminology Criteria for Adverse Events v4.0 (CTCAE).

If grade 1 adverse event occurred, the treatment continued.

In case of grade 2 or higher toxicity the dose was modified according to the potential degree of correlation with the experimental treatment as indicated in the following table:

Toxicity	Relationship with experimental treatment (Tamoxifen/placebo)				
Grade	Unrelated	Unlikely	Possible	Probable	Definite
1	C	C	C	C	C
2	C	C	S-R0	S-R1	S-R1
3	S-R2	S-R2	S-R1	W	W
4	S-R2	S-R1	W	W	W

C = Continue treatment at full dose.

S-R0 = discontinuation of treatment until toxicity reaches grade 1 or lower and restart drug at full dose. If grade 2 toxicity reappears, treatment has to be discontinued again and, once toxicity reaches grade 1, restart drug at 50% reduced dose of (1 tablet every 2 days) until the end of the study.

S-R1 = discontinuation of treatment until toxicity reaches grade 1 or lower, restart drug at 50% reduced dose (1 tablet every 2 days) until the end of the study.

S-R2 = discontinuation of treatment until toxicity reaches grade 1 or lower and restart at 50% reduced dose (1 tablet every 2 days) for one month. If toxicity does not resolve after dose reduction, a further 50% dose reduction (1 tablet every 4 days) is required until the end of the study. No further dose reductions are not allowed and in case of further toxicity the patient has to be discontinued from the study (W).

W = Permanent experimental treatment discontinuation

Concomitant drugs

The concomitant pharmacological treatments were verified at each visit and reported in the appropriate paper form provided by the coordinating center. The prescription of drugs which interfere with the metabolism of tamoxifen: like dicumarolic anticoagulants and the following cytochrome CYP2D6 inhibitor drugs, paroxetine, fluoxetine, sertraline, fluvoxamine, venlafaxine, clomipramine, amitriptyline, cimetidine, fluphenazine, haloperidol, perphenazine, thioridazine was not advised and in any case their use had to be justified and accurately recorded in the specific form.

PHARMACEUTICAL INFORMATION

Study Agents

Generic Name: tamoxifen

Chemical name: (z) -2- [4- (1,2-Dyphenyl-1-butenyl) phenoxy] -N, N-dymethylethanamine citrate

Classification: non-steroidal estrogenic antagonist

CAS Registry No: 10540-29-1

Molecular formula: C26H29NO.C6H8O7

Molecular mass: 563.65

Main chemical characteristics: fine white crystalline powder odorless, melting point 144°C, slightly soluble in water, soluble in methanol, chloroform, acetone, hygroscopic with high relative humidity.

Agent Distribution

Doppel Farmaceutici S.r.l. was responsible for the Batch Release of the Investigational Medicinal Product (IMP) according to the Annex 13 of EU-GMP. The IMP was manufactured and released only after regulatory approval (regulatory agency, IRB) of the protocol and study documentations. The Coordinating Center provided for free each participating site with investigational treatment ready to be given to patients.

Formulation

Tamoxifen tablets: tamoxifen citrate, microcrystalline cellulose, corn starch, povidone, magnesium stearate, croscarmellose sodium, hydroxypropyl methyl cellulose, polyethylene glycol 300, titanium dioxide.

Placebo tablets: microcrystalline cellulose, corn starch, povidone, magnesium stearate, croscarmellose sodium, hydroxypropyl methyl cellulose, polyethylene glycol 300, titanium dioxide.

Packaging and Labeling

Tamoxifen was supplied in bottles of 210 tablets of active compound. The preparation wasvisually identical to that of the placebo.

The treatment was assigned by the Clinical Research Coordination Office in Oncology of the Galliera Hospital. The assignment was double-blind, placebo controlled.

The information on all the label of the experimental drug (tamoxifen or placebo) were: "Study TAM-01 - Period of 6 months: - To be used only for experimental studies - Code number - Batch number - Re-test date - Address and emergency telephone number - Formula (tamoxifen 5mg or placebo) - Dosage (1 tablet / day oral use) - Warnings (Keep out of reach of children) ".

A portion of the label was removable and applied on a special form in order to ensure further control over the correct allocation of the drug.

TRIAL ASSESSMENTS

Instrumental Assessments

A basal bilateral mammogram was mandatory for patient inclusion in the study. Bilateral mammary ultrasound was recommended if the mammogram was dense. At the baseline visit, and for the duration of the study, the Investigator might decide whether to perform transvaginal ultrasound, which was recommended in case of symptoms.

Centralized Laboratory Exams

In a subgroup of subjects blood samples have been taken for the analysis of circulating biomarkers at the baseline visit, at the 12-month visit and the 36-month visit or at the end of the experimental treatment for any reason. Whole blood samples have been collected for determinations of genetic polymorphisms at the baseline visit and at the end of the experimental treatment. Measurements of circulating levels of IGF-I, IGFBP3, SHBG will be performed, and on some SNPs of cytochrome P450 genes involved in the metabolism of tamoxifen CYP2D6 (* 4, * 2, * 5, 6 * and * 7) and CYP2C19. Tissue analysis will also be conducted centrally: evaluation of hormone receptors (estrogen and progesterone) and Her-2, of the proliferative index Ki-67 antigen. The cases considered 2+ for Her-2 (moderate complete membrane immunoreactivity) will be submitted to FISH for confirmation. Tumor tissue microarray and mutation profile by next generation sequencing is also being planned pending additional funding.

For this purpose a centralized blood sample storage and circulating biomarkers analysis laboratory have been set up at the Research Laboratory Section of the Division of Cancer Prevention and Genetics at the European Institute of Oncology. All blood samples have been primarily processed at the recruiting sites in order to be shipped to EIO.

The Division of Cancer Prevention and Genetics at the European Institute of Oncology is also responsible for the centralized genetic polymorphism analysis. The Division has a special section dedicated to clinical cancer genetics, providing also genetic counseling and testing for individual subjects and families. Whole blood samples collected at the recruiting sites have been shipped at the Division and centrally processed.

TRIAL PROCEDURES

Baseline visit

During the baseline visit the mammography and the transvaginal ultrasound were evaluated, if performed, and the first samples for the biomarkers and for the pharmacogenetics were collected. Once the eligibility of the patient was verified she was randomized and the blinded treatment administered. The following data were collected: date of birth, age at menarche, n. children, age at first child, menopausal status, last menstruation, age at menopause, first and second degree familiarity for breast carcinoma and for ovarian cancer, use of hormone therapy, use of alcohol and smoking, previous or current illnesses, weight, height, pubic and umbilical circumference. An objective examination was performed, and the patients were explained the admitted concomitant drugs.

The following data on intraepithelial neoplasia were collected:

Date of surgery, intervention site, type of surgery, sentinel lymph node biopsy, number of previous operations / biopsies / needle aspiration for benign breast condition. Pathological data: histological diagnosis, histotype, presence of comedo, presence of necrosis, stage pT, pN, number of lymph nodes removed, type of neoplasia, multicentric neoplasia, size of the major nodule. Location. Biological markers: estrogen receptors, progesterone receptors, HER2 / neu, KI-67 labeling index.

The vasomotor symptoms and breast cancer prevention trial symptom scale[1] as well as the number and intensity of hot flashes[2] were collected using self administered ad hoc questionnaires.

Evaluations at 6, 12, 18, 24, 30, 36 month visits

A medical interview, a clinic visit and an assessment of possible symptoms and any serious adverse events were repeated every 6 months (± 1 month). An annual breast visit was mandatory, the 6th, 18th and 30th month visits might be performed by clinical or nursing research staff trained under the responsibility of the Investigator.

The mammogram had to be repeated every 12 months to evaluate the effect of treatment. In case of evidence of breast density an ultrasound was recommended.

A gynecological examination and transvaginal ultrasound was performed if indicated and repeated in case of gynecological symptoms, such as bleeding.

Withdrawals for circulating biomarkers were performed only at 12 and 36 month visits or at the end of treatment in case of early termination for other reason.

At each visit the symptom questionnaires were administered to the patient, treatment compliance was verified by pill count and the pill diary completed by the patients, patient also received 6 months (+1) supply kit of drug/placebo.

The following data were collected: date of visit, weight, pubic and umbilical circumference, objective examination assessment, transvaginal ultrasound (performed yes / no, execution date, altered yes / no, endometrial rhyme thickness); mammography (performed yes / no, execution date, altered yes / no); radiotherapy (performed yes / no, start / end date, total dose- only at 6 months visit); treatment compliance (number of tablets consigned/returned, date of); concomitant therapies; evaluation of adverse events had to be reported in the relevant section of the CRF.

Follow-up plan

At the end of the 3 years experimental treatment period the patients has been followed with an annual visit or a telephone contact for at least two more years. During the visit / phone call any symptoms related to the use of the drug and / or a possible onset of any medical event was collected.

Mammography

Incident cases (diagnosis <12 months): mammography were performed before entry into the study. If the patient was randomized within 6 months after surgery and had performed mammograms before surgery, the assessment had not to be repeated for her inclusion in the study.

Prevalent cases (diagnosis> 12 months, <60 months): mammography had to be performed before entry into the study if the previous mammogram was performed after 1 year.

Each center had to provide the Coordinating Centre with both the pre-intervention mammography and the basal one if required.

Follow-up mammography: the control mammograms were performed every 12 months starting from the basal mammogram accordingly the following technical indications

- digital bilateral or analog digitalized mammography,
- recording on hard CD media in DICOM format patient's personal data anonymization.

The collection of images was managed by the Coordinating Center. A detailed technical guide was provided to the enrollment centers for the collection and shipment of the images.

Off-agent criteria

Patients may stop taking study treatment for the following reasons:

- Disease recurrence
- Adverse event or serious adverse event
- Non-compliance
- Physician decision

Patients continued to be followed, if possible, for safety reasons and in order to collect endpoint data according to the schedule of events..

The end of treatment data and the reason for the end of treatment were reported in eCRF.

Off-study criteria

Each patient had the right to withdraw from the trial at any time. In addition, patients may go 'off-study' for the following reasons:

- The protocol intervention and any protocol-required follow-up period is completed.
- Voluntary withdraw of the consent.
- Death.
- Determination of ineligibility (including screen failure).
- Lost to follow-up.

Study Termination

The Sponsor (E.O. Ospedali Galliera) reserves the right to terminate the study at any site and at any time during the study for reasonable medical or administrative reasons. Reasons for study termination may include, but are not limited to, the following:

- Insufficient enrolment.
- Safety concerns.
- Investigator non-compliance with the protocol, GCP, or regulatory requirements.
- Drug supply or manufacturing issue.
- A request to discontinue the study by the local regulatory authorities.

Any premature discontinuation will be appropriately documented according to local requirements (e.g. IRB, regulatory authorities, etc.).

STUDY PROCEDURES FLOW-CHART

Procedures	Baseline	Every 6 months (± 1 month)	Every 12 months (± 1 month)	Drop Out	Follow-up (annual)
Informed consent	X				
Eligibility	X				
Medical history	X				
Hot flashes and BCPT symptoms	X	X	X	X	
Concomitant therapies	X	X		X	X
Randomization	X				
Drug administration	X	X			
Clinical examination	X	X(1)	X	X	(X)
Breast examination	X		X	X	(X)
Mammography	X(2)		X(2)	(X)(2)	(X)
Transvaginal ultrasounds	X(3)	X(3)	(X)(3)	(X)(3)	(X)
Blood biomarkers	X(4)		X	X	
Blood safety tests	X	X		X	X
Medical notes	(X)	(X)	(X)	(X)	X
Symptoms / Adverse reactions	X(5)	X	X	X	X
X = mandatory; (X) = optional, at the discretion of the investigator					

(1) Visits to 6, 18, 30 months may also be performed by trained research nursing staff or clinical researchers coordinated by a study physician
(2) In the case of a dense mammogram, breast ultrasound is recommended
(3) Transvaginal ultrasound is at the discretion of the physician and will be repeated in case of unexpected bleeding
(4) Blood sampling for the drug concentration and biomarkers will be performed at the baseline visit, 12 months, 36 months, in case of early exit from the study for any reason or at the end of the treatment after three years; Whole blood samples for determinations of genetic polymorphisms (genomic DNA) at baseline visit, at the end of treatment (for any reason) and at the end of the study after two years of follow-up
(5) Any pre-existing symptoms should be reported in CRF

STATISTICAL ANALYSIS PLAN

Sample size

According to literature data[3,4], we expect in the placebo group an annual rate incidence of invasive breast cancer and in situ breast ductal carcinoma of about 20/1000. In order to guarantee a power of 80% to detect a 50% reduction (HR = 0.50) of the incidence of breast cancer+DCIS in patients treated with tamoxifen, considering an one-sided alpha level equal to 5% and a drop-out of 10%, a total of 55 events must be observed. With a treatment allocation ratio of 1:1, a 5-year enrollment period and a minimum follow-up period of 2 years, we estimate to recruit 1400 patients in order to observe, at the end of the follow up, the 55 events required. This corresponds to an annual recruitment rate of about 465 women, monthly equal to about 40 women.

The sample size calculation was made with the assumption of a uniform enrollment, in the absence of "time-lag" effect of tamoxifen and hypothesizing a persistence of its effect after the suspension of its administration.

Study population

For the planned statistical analyses, depending on the type of outcome, the following populations will be used:

- Efficacy: Intention-To-Treat (ITT) population: This population includes all randomized patients. All subjects will be included in the treatment group to which they were assigned at the time of randomization, regardless of compliance and the type of treatment actually received. Any randomized subjects mistakenly two or more times will be excluded from the analysis. All women will be followed until the end of the study, even in case of treatment discontinuation.
- Safety: This population includes all randomized patients who received at least one dose of the drug. The treatment group will be defined on the basis of the treatment effectively assumed.

Comparison of characteristics at the time of randomization (“baseline”).

Demographics, clinical and biological characteristics of patients will be described and compared on the basis of their treatment allocation.

Analysis of efficacy

The primary endpoint variable will be the incidence of breast cancer or DCIS (ductal carcinoma in situ).

The main analysis on efficacy will be based on the ITT population. The estimate of the effect will be expressed in terms of hazard ratio, HR (hazard in the placebo group / hazard in the tamoxifen group) and the relative 95% confidence interval. The estimate will be calculated using the semi-parametric Cox proportional hazard (PH) model. The cumulative incidence curves of invasive breast cancer and DCIS using the Kaplan Meier technique will be estimated, log-rank test will be used to test differences between arms and the cumulative probability of developing cancer at 3 years from randomization will be reported, with 95% confidence intervals in order to permit the comparison with risk estimates from literature data.

Subgroup analyses

Main per protocol subgroup analyses will be the following: incident versus prevalent cases, patients with DIN versus LIN, ER+ve versus unknown ER receptor status. A post-hoc comparison of LCIS+ADH versus DCIS although not specifically planned will be tested as well.

Prior to proceed with the subgroup analyses, we will use the semi-parametric Cox PH model to study the interaction effect between treatment and each stratification factor. Only in case of a statistically significant interaction term (defined as a p-value $\leq$ 10%, two-tailed test) it will be possible to estimate the effect (HR) in each stratum identified by the stratification factor.

All subgroup analyzes will be performed on the ITT population.

REFERENCES

1. Stanton AL, Bernaards CA, Ganz PA. The BCPT Symptom Scales: a measure of physical symptoms for women diagnosed with or at risk for breast cancer. Journal of the National Cancer Institute 2005; 97:448-456.
2. Sloan A, Loprinzi CL, Novotny PL, et al. Methodologic Lessons Learned From Hot Flash Studies. J Clin Oncol 2001;19:4280-4290.
3. Fisher B, Costantino JP, Wickerham DL, et al Tamoxifen for prevention of breast cancer: report of the National Surgical Adjuvant Breast and Bowel Project P-1 Study. J Natl Cancer Inst. 1998;90:1371-88.
4. Fisher B, Dignam J, Wolmark N, et al Tamoxifen in treatment of intraductal breast cancer: National Surgical Adjuvant Breast and Bowel Project B-24 randomised controlled trial. Lancet. 1999 ;353:1993-2000.

SUPPLEMENTARY MATERIAL 3

TC Report IDMC and REC progress report

Ente Ospedaliero
Ospedali Galliera
Genova
Ospedale di rilievo nazionale e di alta specializzazione

Livello 2 Medico
Dipartimento: area di medicina

S. C. Oncologia medica

Dirigente responsabile: Prof. Andrea De Censi

June 28, 2018

Teleconference Report June 28, 2018 Independent Data Monitoring Committee (IDMC) of the study: "Randomized placebo controlled phase III trial with low-dose tamoxifen in women with intraepithelial breast cancer - TAM-01"

Participants:

Alberto Costa (IDMC Chair)
Sara Gandini (IDMC)
Paolo Pronzato (IDMC)
Andrea De Censi (PI)
Matteo Puntoni (Statistician of the Study)
Silvia Caviglia (Study Coordinator - Secretary of the minutes of the teleconference)

The IDMC evaluated the pros and cons of submitting an abstract to the San Antonio Breast Cancer Symposium 2018 (deadline 3/7). A teleconference was held to discuss the opportunity to proceed with the publication of the data from a preliminary interim analysis, not planned in the protocol but which will be the subject of a formal communication to the Regional Ethics Committee.

The decision to carry out the analysis, although not planned, is based on four main reasons:

1) the mature follow-up accumulated by the cohort (median follow-up: 5.1 years);

2) all 500 recruited women have completed the 3 years of treatment;

3) a total of 43 events occurred, corresponding to 80% of the expected events (55 events);

4) adverse events were lower than expected.

The summary of the efficacy and toxicity open-label analysis performed by the study statisticians shows that:

- At 31 May 2018, after a median follow-up of 5.1 years (interquartile range 3.9-6.3), 14 events occurred in the arm of treatment with Tamoxifen (T) and 28 in the arm with Placebo (P) (Hazard Ratio = 0.48, 95% CI, 0.25-0.90, p = 0.02). There is also an additional event due to a MI death in the placebo arm.

OG
Mura delle Cappuccine 14 - 16128 Genova - Tel. +39 01056321 - Fax 010 563 2018 - www.galliera.it
P.I. 00557720109 - Cod. IBAN IT 80 T 06175 01590 000000414190 - protocollo@pec.galliera.it

- The incidence rate of events was 11.8 per 1000 person-years (py) in the T arm and 24.9 per 1000 py in the P arm

- Most recurrences were invasive cancers of the breast: 11/14 (78%) in the T arm and 16/29 (55%) in the P arm

- There were 8 serious adverse events in the T arm and 12 in the P arm, including 2 arterial events in each arm, 2 superficial phlebitis in the T arm and 1 endometrial cancer (annual rate 0.85 per 1000 py) in the T arm

- In the arm P and T, 6 and 4 seconds primary tumors occurred, 2 died in the P arm

- Menopausal symptoms appear more frequently in the T-arm but no statistical analysis has been performed on this aspect so far. The data will be analyzed in more detail as soon as possible.

Dr. Costa and Dr. Pronzato express a favorable opinion to the publication of these results above all for an ethical motivation. It is considered important and responsible to inform the women who participated in the trial and the scientific community of the favorable effect of tamoxifen at the dosage of 5 mg per day both in terms of efficay and safety.

Dr. Gandini agrees with this opinion and believes that the results may be considered "mature" for a publication and that it is quite unlikely that, by extending the follow-up further, this statistical significance will be rejected.

The Data Safety Monitoring Board therefore authorizes the publication of an interim analysis.

The decision is made to proceed with a formal communication to the REC that includes an interim analysis for the reasons explained above.

Sincerely,

Silvia Caviglia

Mura delle Cappuccine 14 - 16128 Genova - Tel. +39 01056321 - Fax 010 563 2018 - www.galliera.it
P.I. 00557720109 - Cod. IBAN IT 80 T 06175 01590 000000414190 - protocollo@pec.galliera.it

Ente Ospedaliero
Ospedali Galliera
Genova
Ospedale di rilievo nazionale e di alta specializzazione

Livello 2 Medico
Dipartimento: area di medicina

S. C. Oncologia medica

Dirigente responsabile: Prof. Andrea De Censi

Subject: Progress report on the "Randomized placebo-controlled phase III trial with low-dose tamoxifen in women with breast intraepithelial neoplasia (IEN) - TAM-01" EUDRACT NUMBER: 2007-007740-10

24.09.2018

To Regional Ethical Committee - Liguria

Dear Sirs,

please find enclosed the report on the state of the art of the above mentioned study.

The study is coordinated by the Division of Medical Oncology of the E.O. Galliera Hospital, Principal Investigator Prof. Andrea De Censi. Currently there are 14 participating centers (Emendment 6. Version 2 of 20 January 2014) with a total of 500 patients being enrolled as of 27/02/2015, 360 of whom agreed to participate in the pharmacogenetics study.
On February 27, 2015 the enrollment was closed.

Table 1 <u>Recruitment summary</u>

Status: Closed

Start : November 12, 2008

Closing: February 28, 2015

Enrolled patients: 500

Mura delle Cappuccine 14 - 16128 Genova - Tel. +39 01056321 - Fax 010 563 2018 - www.galliera.it
P.I. 00557720109 - Cod. IBAN IT 80 T 06175 01590 000000414190 - protocollo@pec.galliera.it

Table 2 Participating Centers and patients enrolled per center

Country	Center	Opening date	Closing date	Accrual duration	Registered patients	Monthly accrual rate
ITALIA	BARI [D'AMICO]	08-FEB-2010	--	1905	32	0.511
	CAGLIARI [CABULA]	01-SEP-2011	24-DEC-2012	481	0	0.000
	CARPI [CAGOSSI]	08-SEP-2011	--	1328	12	0.275
	CATANZARO [DI PAOLA]	09-SEP-2010	--	1692	6	0.108
	FORLI [FALCINI]	20-JAN-2011	--	1559	39	0.760
	GENOVA IST [CANAVESE]	29-JUL-2009	24-DEC-2012	1245	3	0.073
	GENOVA [DECENSI]	01-JUN-2008	--	2522	7	0.084
	GENOVA [FRIEDMAN]	03-MAY-2010	24-DEC-2012	967	2	0.063
	LUGO [CRUCIANI]	02-FEB-2011	--	1546	30	0.590
	MILANO HUMANITAS [TINTERRI]	10-DEC-2010	24-DEC-2012	746	0	0.000
	MILANO IEO [BONANNI]	02-SEP-2009	--	2064	117	1.723
	MODENA [CORTESI]	02-FEB-2010	--	1911	56	0.923
	NAPOLI [D'AIUTO]	16-NOV-2009	--	1989	60	0.917
	PAVIA [PEGOLO]	01-SEP-2011	--	1335	5	0.114
	TORINO [PONTI]	21-NOV-2009	--	1984	46	0.705
	TORTONA [PACQUOLA]	06-AUG-2010	--	1726	40	0.705
	VARESE [GIARDINA]	03-JUN-2010	--	1790	12	0.204
	VICENZA [GULISANO]	06-SEP-2010	--	1695	34	0.610
Total				28485	503	0.537

Study treatment

In 2014, the coordinating center required the production of a new batch of experimental drug (tamoxifen 5 mg) and matching placebo in order to guarantee study treatment for all patients who would have completed the 3 years period at the beginning of 2018. The length of the study was prolonged due to the delayed activation phase of the centers involved in the trial and this delay was mainly due to financial and administrative issues. The Coordinating Centre together with the Pharmacy, E.O. Galliera Hospital selected Doppel Farmacutici S.r.l. Cortemaggiore (PC), a company who is conforming to Good Manufacturing Practices (GMP) and is accredited by the Italian Medicines Agency (AIFA) for the production of the experimental drug and placebo. This company had already been the supplier of tamoxifen and placebo in 2012 and 2013.

In March 2018, all patients completed the experimental treatment.

OG Mura delle Cappuccine 14 - 16128 Genova - Tel. +39 01056321 - Fax 010 563 2018 - www.galliera.it
P.I. 00557720109 - Cod. IBAN IT 80 T 06175 01590 000000414190 - protocollo@pec.galliera.it

Independent Data Monitoring Committee meetings (IDMC)

The IDMC reviewed the state of the study annually. Below we summarize the most relevant meetings. On November 28, 2013 the Independent Data Monitoring Committee whose members are: Dr. Alberto Costa, MD (Surgical Oncologist, Italian Senology Center of Switzerland, Switzerland, Committee Chairman), Dr. Sara Gandini, PhD (Biostatistician, European Institute of Oncology, Milan) and Dr. Paolo Pronzato, MD (Medical Oncologist, AUO Ist-San Martino Hospital, Genoa) held a meeting. In addition to the members of the IDMC Andrea De Censi (Principal Investigator) and Matteo Puntoni (Study Biostatistic) participated in the discussion. All serious adverse events (SAEs) were reviewed and the IDMC members agreed that no unexpected safety issues were identified. Due to financial constraints (the Ministry of Health stopped funding all the studies included in the program named "Tevere" and the LILT quit the funding as well), the IDMC advised to close the enrollment to 500 women. At the time of the meeting a total of 10 events (recurrent breast cancer) were observed. Therefore the study did not have the statistical power needed to evaluate the hypothesized risk reduction effect (HR = 0.5).

On May 5, 2017, the IDMC recommended to update the patients follow-up to increase the study maturity. There were no outstanding safety issues. The IDMC recommended the Steering Committee to carry out a review of the events defined by the study primary endpoint, as well as of the follow-up status.

On June 28, 2018, the IDCM evaluated the progress of the trial and recommended the disclosure of the results and the presention to an important international meeting. This decision was based on ethical considerations and in light of a potential change in clinical practice. The following points were taken into account: the study had more than 5 years of median follow-up (5.1 years, interquartile range: 3.9-6.3); all patients had completed 3 years of treatment and 80% (n = 43) of expected events had been recorded; the toxicity was lower than expected.

The IDMC therefore asked the study statistician to carry out an unblinded interim analysis of the efficacy and toxicity results, even though it was not initially foreseen by the protocol.

The analysis showed that: 1) the relapse rate in the placebo arm was significantly higher than expected; 2) the effect of tamoxifen was a 52% risk reduction in relapse, and 3) compared to the dose of 20 mg daily there was an indirect but clear evidence for a lower risk of serious adverse events. Based on this evidence, the study's IDCM recommended the publication of the trial data as they are potentially relevant to clinical practice and do not compromize the protection of participating women still in follow-up.

Below, further descriptive data on the study cohort:

OG Mura delle Cappuccine 14 - 16128 Genova - Tel. +39 01056321 - Fax 010 563 2018 - www.galliera.it
P.I. 00557720109 - Cod. IBAN IT 80 T 06175 01590 000000414190 - protocollo@pec.galliera.it

Table 3 ***Serious Adverse Event (SAE)***

paz id	Description	Related
7	Istero-anessiectomy (ovarian mucinous cystadenoma and endocervical polyp)	N
8	Superficial phlebitis (saphenectomy)	Y
35	*Thyroid cancer*	
53	Colic for gallbladder stones (cholecystectomy)	N
67	Acute headache (angioma venoso temporale sx + ernia discale c5-c6)	N
115	*Ovarian cancer*	
117	Acute coronary syndrome (IMA & ACS)	N
121	*Parotid carcinoma*	
125	Acute coronary syndrome	N
143	*Cervical cancer*	
144	Pneumonitis	N
164	Gram-negative bacteria infections	N
164	*Lymphoma*	
176	*Bone marrow cancer*	
200	*Ovarian cancer (surgery)*	
222	Deep vein thrombosis	Y
250	Pneumonitis	N
258	Acute coronary syndrome	N
304	*Endometrial cancer*	
307	*Adenocarcinoma of the appendix; peritoneal carcinosis*	

Mura delle Cappuccine 14 - 16128 Genova - Tel. +39 01056321 - Fax 010 563 2018 - www.galliera.it
P.I. 00557720109 - Cod. IBAN IT 80 T 06175 01590 000000414190 - protocollo@pec.galliera.it

356	*Kidney cancer*	
376	*Basal cell carcinoma*	
404	Duodenal diverticular perforation and abdominal abscess	N
452	Depressed level of consciousness	N
482	Tibia fracture	N

Table 4 Baseline preliminary data of the characteristics of the patients from the unblinded analysis requested by the IDMC

Total randomized patients **n = 503** Patients randomized by mistake: **n = 3** (id: 45.183,218)

Table 1. Main subject and tumor characteristics.

	Tamoxifen N=253	Placebo N=247
Age (yrs), *mean (SD)*	54 (9.6)	54 (9.1)
Menopausal status, n (%)		
Pre-	113 (45)	108 (44)
Post-	136 (55)	137 (56)
Age at menopause (yrs), *mean (SD)*	50 (4.5)	49 (5.9)
Body mass index (Kg/m^2), *mean (SD)*	25.7 (4.8)	25.3 (4.2)
Waist to Hip ratio, *mean (SD)*	0.86 (0.1)	0.86 (0.1)
Endometrial thickness (mm), *mean (SD)*	5.4 (4.7)	5.0 (4.3)
1st/2nd degree breast/ovarian cancer FH, n (%)	94 (37)	95 (38)
Histological type, *n (%)*		
ADH (DIN 1b)	51 (21)	49 (20)
LCIS (LIN 2-3)	26 (10)	25 (10)
DCIS grade 1 (DIN 1c)	42 (17)	40 (16)
DCIS grade 2 (DIN 2)	71 (29)	87 (36)
DCIS grade 3 (DIN 3)	58 (23)	44 (18)
Type of surgery, *n (%)*		
Conservative (Q/L)	209 (84)	201 (82)
Mastectomy	39 (16)	44 (18)
Axillary dissection	1 (0)	-

Mura delle Cappuccine 14 - 16128 Genova - Tel. +39 01056321 - Fax 010 563 2018 - www.galliera.it
P.I. 00557720109 - Cod. IBAN IT 80 T 06175 01590 000000414190 - protocollo@pec.galliera.it

Comedo, *n (%)*	27 (11)	24 (10)
Necrosis, *n (%)*	63 (26)	60 (25)
Multifocal neoplasia, *n (%)*	71 (29)	67 (27)
Radiotherapy, *n (%)*	106 (42)	106 (42)

Abbreviations: SD=Standard deviation, FH=family history.

Abbreviations: ADH=Atypical Ductal Hyperplasia, DIN=Ductal Intraepithelial Neoplasia, LIN=Lobular Intraepithelial Neoplasia, IQR=Interquartile range.

We look forward to receiving your feed back in due course.

Sincerely,

Andrea De Censi

Andrea De Censi

Mura delle Cappuccine 14 - 16128 Genova - Tel. +39 01056321 - Fax 010 563 2018 - www.galliera.it
P.I. 00557720109 - Cod. IBAN IT 80 T 06175 01590 000000414190 - protocollo@pec.galliera.it

SUPPLEMENTARY DATA SET
