# Supplementary material for: Association of CYP2D6 genotype and tamoxifen metabolites with breast cancer recurrence in a low-dose trial
Source: NPJ Breast Cancer. 2021 Mar 25;7:34. doi: 10.1038/s41523-021-00236-6 (PMC7994552; doi:10.1038/s41523-021-00236-6)
Supplement: Supplementary file 3 — Reporting Summary Checklist [file 41523_2021_236_MOESM3_ESM.pdf]

nature research

Corresponding author(s): DBPR - NPJBCANCER-00920

Last updated by author(s): 01/25/2021

# Reporting Summary

Nature Research wishes to improve the reproducibility of the work that we publish. This form provides structure for consistency and transparency in reporting. For further information on Nature Research policies, see our Editorial Policies and the Editorial Policy Checklist.

## Statistics

For all statistical analyses, confirm that the following items are present in the figure legend, table legend, main text, or Methods section.

| n/a | Confirmed | |
|---|---|---|
| ☐ | ☒ | The exact sample size (*n*) for each experimental group/condition, given as a discrete number and unit of measurement |
| ☐ | ☒ | A statement on whether measurements were taken from distinct samples or whether the same sample was measured repeatedly |
| ☐ | ☒ | The statistical test(s) used AND whether they are one- or two-sided<br>*Only common tests should be described solely by name; describe more complex techniques in the Methods section.* |
| ☐ | ☒ | A description of all covariates tested |
| ☐ | ☒ | A description of any assumptions or corrections, such as tests of normality and adjustment for multiple comparisons |
| ☐ | ☒ | A full description of the statistical parameters including central tendency (e.g. means) or other basic estimates (e.g. regression coefficient) AND variation (e.g. standard deviation) or associated estimates of uncertainty (e.g. confidence intervals) |
| ☐ | ☒ | For null hypothesis testing, the test statistic (e.g. *F*, *t*, *r*) with confidence intervals, effect sizes, degrees of freedom and *P* value noted<br>*Give P values as exact values whenever suitable.* |
| ☒ | ☐ | For Bayesian analysis, information on the choice of priors and Markov chain Monte Carlo settings |
| ☒ | ☐ | For hierarchical and complex designs, identification of the appropriate level for tests and full reporting of outcomes |
| ☐ | ☒ | Estimates of effect sizes (e.g. Cohen's *d*, Pearson's *r*), indicating how they were calculated |

*Our web collection on statistics for biologists contains articles on many of the points above.*

## Software and code

Policy information about availability of computer code

| | |
|---|---|
| Data collection | data collection was performed using a non commercial web-based software platform based on SAS software (https://www.eclintrials.org/) |
| Data analysis | all statistical analyses were performed using the software STATA (StataCorp. 2015. Stata Statistical Software: Release 14. College Station, TX: StataCorp LP.) |

For manuscripts utilizing custom algorithms or software that are central to the research but not yet described in published literature, software must be made available to editors and reviewers. We strongly encourage code deposition in a community repository (e.g. GitHub). See the Nature Research guidelines for submitting code & software for further information.

## Data

Policy information about availability of data

All manuscripts must include a data availability statement. This statement should provide the following information, where applicable:

- Accession codes, unique identifiers, or web links for publicly available datasets
- A list of figures that have associated raw data
- A description of any restrictions on data availability

Andrea DeCensi and Matteo Puntoni had full access to all the data in the study and take responsibility for the integrity of the data and the accuracy of the data analysis. Data may be shared upon request for collaborative studies.

# Field-specific reporting

Please select the one below that is the best fit for your research. If you are not sure, read the appropriate sections before making your selection.

☒ Life sciences ☐ Behavioural & social sciences ☐ Ecological, evolutionary & environmental sciences

For a reference copy of the document with all sections, see nature.com/documents/nr-reporting-summary-flat.pdf

# Life sciences study design

All studies must disclose on these points even when the disclosure is negative.

| | |
|---|---|
| Sample size | This is a secondary publication of the trial, the sample size calculation was performed and described in the main paper which is referenced in the methods section. |
| Data exclusions | As described in the text and in the main paper, measures of serum biomarkers/tamoxifen metabolites levels and CYP2D6 genotyping were performed in a subgroup of patients of the main trial. We have not excluded any data. |
| Replication | These are secondary results of a randomized clinical trial, which is the gold standard for clinical research. Data replication could only be possible by carrying out another study identical to this one which would not be ethical. |
| Randomization | Randomization was performed and described in the main paper which is referenced in the methods section. |
| Blinding | Blinding was performed and described in the main paper which is referenced in the methods section. |

# Reporting for specific materials, systems and methods

We require information from authors about some types of materials, experimental systems and methods used in many studies. Here, indicate whether each material, system or method listed is relevant to your study. If you are not sure if a list item applies to your research, read the appropriate section before selecting a response.

## Materials & experimental systems

| n/a | Involved in the study | |
|---|---|---|
| ☒ | ☐ | Antibodies |
| ☒ | ☐ | Eukaryotic cell lines |
| ☒ | ☐ | Palaeontology and archaeology |
| ☒ | ☐ | Animals and other organisms |
| ☐ | ☒ | Human research participants |
| ☐ | ☒ | Clinical data |
| ☒ | ☐ | Dual use research of concern |

## Methods

| n/a | Involved in the study | |
|---|---|---|
| ☒ | ☐ | ChIP-seq |
| ☒ | ☐ | Flow cytometry |
| ☒ | ☐ | MRI-based neuroimaging |

## Human research participants

Policy information about studies involving human research participants

| | |
|---|---|
| Population characteristics | Women75 years of age or younger with an Eastern Cooperative Oncology Group performance status of 1 or less and operated hormone-sensitive (ER or progesterone receptor >= 1%) or unknown breast intraepithelial neoplasia (ADH,DCIS, or LCIS). Women with high-grade or comedo/necrotic DCIS received adjuvant radiotherapy of 50 Gy in 25 courses. Women were visited every 6 months and had a mammography and transvaginal ultrasound annually for 3 years of treatment and 2 years of follow-up. Main exclusion criteria were any prior cancer, any tamoxifen contraindications, mental disorders, pregnancy, grade 2 or higher biochemical alterations according to theNational Cancer Institute Common Terminology Criteria for Adverse Events (version 4), prior use of anti-estrogens,current use of dicumarols, and CYP2D6 inhibitors suchas selective serotonin reuptake inhibitors. |
| Recruitment | Between November 1, 2008, and March 31, 2015, 1,160 women were screened and 500 women 75 years of age or younger were included in the study. |
| Ethics oversight | The study(Tam01) was approved by the ethics committee of the sponsor (Galliera Hospital, Genoa, Italy) and participating sites, and all women signed a written informed consent. |

Note that full information on the approval of the study protocol must also be provided in the manuscript.

## Clinical data

Policy information about clinical studies

All manuscripts should comply with the ICMJE guidelines for publication of clinical research and a completed CONSORT checklist must be included with all submissions.

| | |
|---|---|
| Clinical trial registration | ClinicalTrials.gov Identifier: NCT01357772; https://clinicaltrials.gov/ct2/show/NCT01357772 |
| Study protocol | The study protocol can be viewed in Supplementary Material 2-3 |
| Data collection | We conducted a multicenter phase III trial of tamoxifen 5 mg/d versus placebo administered for 3 years in women 75 years of age or younger. All participating centers were Italian. Patients were randomly assigned (1:1) to one of the two treatment arms in a masked fashion by a Web-based system using the minimization algorithm. Recruitment began in November 1, 2008 and was closed in March 31, 2015. Women were visited every 6 months and had a mammography and transvaginal ultrasound annually for 3 years of treatment and 2 years of follow-up. Blood draws for secondary end-points were drawn at baseline, and at each annual visit (or at study cessation). |
| Outcomes | The primary end point was the incidence of invasive breastcancer or ductal carcinoma in situ. Secondary end points were incidence of atypical ductal hyperplasia or lobular carcinoma in situ, endometrial cancer, other second primary cancers, deep venous thromboembolic events, coronaryheart disease, bone fractures, cataract, and menopausal symptoms. Correlative studies of biomarkers included mammographic density, circulating insulin-like growth factor (IGF)-I and IGF binding protein-3, sex hormone binding globulin, C-reactive protein, CYP2D6 single nucleotide polymorphisms, tamoxifen, and metabolite blood levels. |
